# Speech Auditory Brainstem Responses: Effects of Background, Stimulus Duration, Consonant–Vowel, and Number of Epochs

**DOI:** 10.1097/AUD.0000000000000648

**Published:** 2019-04-26

**Authors:** Ghada BinKhamis, Agnès Léger, Steven L. Bell, Garreth Prendergast, Martin O’Driscoll, Karolina Kluk

**Affiliations:** 1 Manchester Centre for Audiology and Deafness, Division of Human Communication, Development & Hearing, School of Health Sciences, Faculty of Biology, Medicine and Health, Manchester Academic Health Science Centre, University of Manchester, United Kingdom; 2 Department of Communication and Swallowing Disorders, King Fahad Medical City, Riyadh, Saudi Arabia; 3 Signal Processing and Control Group, Institute of Sound and Vibration Research, University of Southampton, Southampton, United Kingdom; 4 Manchester Auditory Implant Centre, Central Manchester University Hospitals NHS Foundation Trust, Manchester, United Kingdom.

**Keywords:** Consonant–vowel, Noise, Quiet, Speech-ABR, Stimulus Duration

## Abstract

**Objectives:**

The aims of this study were to systematically explore the effects of stimulus duration, background (quiet versus noise), and three consonant–vowels on speech-auditory brainstem responses (ABRs). Additionally, the minimum number of epochs required to record speech-ABRs with clearly identifiable waveform components was assessed. The purpose was to evaluate whether shorter duration stimuli could be reliably used to record speech-ABRs both in quiet and in background noise to the three consonant–vowels, as opposed to longer duration stimuli that are commonly used in the literature. Shorter duration stimuli and a smaller number of epochs would require shorter test sessions and thus encourage the transition of the speech-ABR from research to clinical practice.

**Design:**

Speech-ABRs in response to 40 msec [da], 50 msec [ba] [da] [ga], and 170 msec [ba] [da] [ga] stimuli were collected from 12 normal-hearing adults with confirmed normal click-ABRs. Monaural (right-ear) speech-ABRs were recorded to all stimuli in quiet and to 40 msec [da], 50 msec [ba] [da] [ga], and 170 msec [da] in a background of two-talker babble at +10 dB signal to noise ratio using a 2-channel electrode montage (Cz-Active, A1 and A2-reference, Fz-ground). Twelve thousand epochs (6000 per polarity) were collected for each stimulus and background from all participants. Latencies and amplitudes of speech-ABR peaks (V, A, D, E, F, O) were compared across backgrounds (quiet and noise) for all stimulus durations, across stimulus durations (50 and 170 msec) and across consonant–vowels ([ba], [da], and [ga]). Additionally, degree of phase locking to the stimulus fundamental frequency (in quiet versus noise) was evaluated for the frequency following response in speech-ABRs to the 170 msec [da]. Finally, the number of epochs required for a robust response was evaluated using *F*_sp_ statistic and bootstrap analysis at different epoch iterations.

**Results:**

Background effect: the addition of background noise resulted in speech-ABRs with longer peak latencies and smaller peak amplitudes compared with speech-ABRs in quiet, irrespective of stimulus duration. However, there was no effect of background noise on the degree of phase locking of the frequency following response to the stimulus fundamental frequency in speech-ABRs to the 170 msec [da]. Duration effect: speech-ABR peak latencies and amplitudes did not differ in response to the 50 and 170 msec stimuli. Consonant–vowel effect: different consonant–vowels did not have an effect on speech-ABR peak latencies regardless of stimulus duration. Number of epochs: a larger number of epochs was required to record speech-ABRs in noise compared with in quiet, and a smaller number of epochs was required to record speech-ABRs to the 40 msec [da] compared with the 170 msec [da].

**Conclusions:**

This is the first study that systematically investigated the clinical feasibility of speech-ABRs in terms of stimulus duration, background noise, and number of epochs. Speech-ABRs can be reliably recorded to the 40 msec [da] without compromising response quality even when presented in background noise. Because fewer epochs were needed for the 40 msec [da], this would be the optimal stimulus for clinical use. Finally, given that there was no effect of consonant–vowel on speech-ABR peak latencies, there is no evidence that speech-ABRs are suitable for assessing auditory discrimination of the stimuli used.

## Abstract 2

Supplemental Digital Content is available in the text.

## INTRODUCTION

The auditory brainstem response (ABR) is an auditory evoked potential that is recorded from the scalp in response to multiple short auditory stimuli such as clicks, tone bursts, or chirps (Hall 2015). The ABR to clicks and tone-bursts is a well-established clinical measure that is widely used to evaluate hearing in patients that are unable to perform standard behavioral hearing threshold measures. The ABR has advantages over other auditory evoked potentials in that it is not influenced by attention or state of arousal and that the response can be reliably recorded from infants and young children (Hall 2015; Hood 2015). The ABR could also be measured in response to short consonant–vowel (CV) stimuli (e.g., [ba] [da] [ga]; Skoe & Kraus 2010). This type of ABR will be referred to as the speech-ABR. It has been shown that the speech-ABR waveform follows the temporal and spectral features of the CV stimulus; these features play an important role in speech understanding in that (1) onset of sound facilitates phoneme identification; (2) frequency transitions allow consonant identification; (3) formant structure facilitates vowel identification; and (4) the fundamental frequency (F_0_) portrays nonlinguistic information such as sex and emotion (Kraus & Nicol 2005; Abrams & Kraus 2015). These temporal and spectral features of speech cannot be measured through current clinical ABRs to click and tone burst stimuli. It has, therefore, been proposed that the speech-ABR may be used as a measure of (1) brainstem speech encoding (e.g., Kraus & Nicol 2005; Johnson et al. 2005; Chandrasekaran & Kraus 2010); (2) speech-in-noise performance, where responses in noise are more degraded with longer peak latencies and smaller peak amplitudes than responses in quiet and are more degraded in individuals who perform worse on behavioral speech-in-noise measures compared with those who perform better (e.g., Anderson et al. 2011; Parbery-Clark et al. 2011; Song et al. 2011b); and (3) auditory discrimination of different CVs, where CVs with a higher second formant (F_2_) frequency have shorter peak latencies than CVs with a lower F_2_ frequency (e.g., Johnson et al. 2008; Hornickel et al. 2009b). Therefore, the speech-ABR may have potential for clinical application in audiology as an objective measure of detection of speech sounds, speech-in-noise performance, and discrimination of different speech sounds. The speech-ABR could compliment currently available clinical ABRs that were introduced into audiology clinical practice in the 1980s (Galambos & Despland 1980) after a period of lab-based investigations since the discovery of ABRs in 1970 (Jewett et al. 1970). The reader is referred to Hall 2015 (chapters 4 and 5) for a review of the transition of current clinical ABRs from research to clinical practice.

The length of CV stimuli used in the literature ranges from short (no sustained vowel period), to long, for example, 40 msec (e.g., Hornickel et al. 2009a; Krizman et al. 2010), 60 msec (e.g., Akhoun et al. 2008), 170 msec (e.g., Johnson et al. 2008; Song et al. 2011b), and 180 msec (e.g., Bellier et al. 2015). The shorter CV (40 msec) contains an onset burst and a formant transition period without a sustained vowel period. Subsequently, speech-ABRs to the 40 msec [da] contain onset peaks (V and A), transition peaks (D, E, and F), and offset peak (O; e.g., Hornickel et al. 2009a). The longer CVs (170 and 180 msec) contain an onset burst, a formants transition period, and a sustained vowel period. Subsequently, speech-ABRs to longer CVs contain onset and transition peaks and an additional frequency following response (FFR; e.g., Johnson et al. 2008; Bellier et al. 2015).

Researchers who used the speech-ABR to assess speech-in-noise performance mainly used the 170 msec [da] (e.g., Anderson et al. 2011; Parbery-Clark et al. 2011; Song et al. 2011a,b; Hornickel et al. 2012), while the 40 msec [da] was used only by a few (e.g., Russo et al. 2004; Anderson et al. 2013a). Additionally, 170 msec [ba] [da] [ga] were researched in the context of evaluating discrimination between CVs via the speech-ABR (e.g., Johnson et al. 2008; Hornickel et al. 2009b). The rationale behind selecting longer stimuli over shorter stimuli for speech-ABRs in noise and for speech-ABRs to different CVs has not been discussed in the literature. While the use of longer stimuli that contain a sustained vowel period or a vowel with changing pitch trajectories would be necessary to assess certain populations such as native speakers of tonal languages (e.g., Krishnan et al. 2005; Swaminathan et al. 2008) or individuals with autism spectrum disorder (e.g., Russo et al. 2008), shorter stimuli may be appropriate to elicit speech-ABRs in noise and speech-ABRs to different CVs. We postulate that longer stimuli are commonly used because they have a closer resemblance to natural speech and their speech-ABRs contain a sustained period (FFR) that would result in responses that contain more components than responses to shorter stimuli. However, longer stimuli would require longer recording sessions, which may hinder the speech-ABRs’ clinical applicability. Nonetheless, the effect of stimulus duration on the speech-ABR in noise and the speech-ABR to different CVs has not yet been assessed.

The speech-ABR has the potential to become a clinical audiological measure. However, stimulus duration would influence the implementation of the speech-ABR in the clinical setting. Specifically, shorter stimuli would be more clinically feasible as they would require shorter recording sessions. Shorter stimuli have been used to record speech-ABRs in noise and thus may have potential use in assessing speech-in-noise performance with the speech-ABR (e.g., Russo et al. 2004; Anderson et al. 2013a). With regard to the use of speech-ABRs to assess discrimination between CVs, shorter stimuli may be sufficient to record speech-ABRs if the difference in F_2_ frequency between CVs is reflected in the vowel formant transition period for each CV. Additionally, the method used to analyze discrimination between CVs should not require the sustained period as a control condition as is required in cross-phasogram analysis (e.g., Skoe et al. 2011). Another factor that may influence the clinical implementation of the speech-ABR is the minimum number of epochs (number of repetitions) required to obtain a response with clearly identifiable waveform components (peaks). A larger number of epochs requires longer recording sessions. Number of epochs used in speech-ABR literature ranges from 4000 to 6000 (e.g., Johnson et al. 2008; Hornickel et al. 2009a; Skoe & Kraus 2010; Skoe et al. 2015). However, the minimum number of epochs required to obtain speech-ABRs with clearly identifiable peaks has not yet been addressed.

The aim of this study was to assess the effect of background (quiet versus noise) and stimulus duration on speech-ABRs. Speech-ABR time domain waveforms evoked by 3 CVs ([ba] [da] [ga]) of short duration (40 and 50 msec) and long duration (170 msec) in two backgrounds (quiet and noise) were evaluated in order to (1) assess whether short CVs can be reliably used to measure speech-ABRs in quiet and in noise; (2) evaluate the differences in responses to short versus long CVs; and (3) determine whether auditory discrimination between CVs ([ba], [da], [ga]) can be assessed with short CVs. The issue of the minimum number of epochs required to obtain a speech-ABR with clearly identifiable peaks was also addressed.

## MATERIALS AND METHODS

### Participants

Twelve adults (aged 22 to 49 years; mean = 31.42; SD = 7.88; 7 females) with normal hearing (≤25 dBHL at 250 to 8000 Hz), normal click-ABRs at 100 dB peak equivalent SPL (peak latencies (ms); I: mean = 1.86, SD: 0.18, III: mean = 4.00, SD = 0.19, V: mean = 5.89, SD = 0.21), and no history of neurological disorders or learning difficulties were tested. Participants were recruited from the University of Manchester and were compensated for their time. All participants provided written informed consent before enrolment in this study.

This study was approved by the University of Manchester research ethics committee (Ref: UREC 15487).

### Speech-ABR Recording

#### Equipment

Raw EEG responses were collected with Cambridge Electronic Design (CED, Cambridge, UK) “Signal” software (version 5.11) using a CED power 1401 mkII data acquisition interface (CED Limited) and a Digitimer 360 isolated 8-channel patient amplifier (Digitimer Limited, Hertfordshire, UK). Speech-ABR stimuli were presented from the CED Signal software through the CED power 1401 mkII and routed through a Tucker-Davis Technologies (TDT, Alachua, FL) PA5 Programmable attenuator and a TDT HB7 Headphone Driver to E.A.RTONE 3A insert earphones (E.A.R Auditory Systems, Aearo Company, Indianapolis, IN). Background noise was presented from Audacity (version 1.2.6) via an E-MU 0202 sound card (Creative Technology Limited, UK) and routed through the TDT HB7 Headphone Driver to the E.A.RTONE 3A insert earphones; splitters were used in order for the stimuli and noise to be presented through the same insert earphone. Stimuli (CVs and background noise) were calibrated in dB A using a Brüel and Kjær type 2250 (Brüel and Kjær, Nærum, Denmark) sound-level meter.

#### Stimuli

Three stimulus durations were used: (1) 5-formant synthesized 40 msec [da] (described in Banai et al. 2009); (2) 6-formant synthesized 50 msec [ba] [da] and [ga]; and (3) 6-formant synthesized 170 msec [ba] [da] and [ga] (described in Hornickel et al. 2009b). The 40 msec [da] and the 170 msec CVs ([ba] [da] [ga]) are identical to those used in the literature; however, the 50 msec CVs ([ba] [da] [ga]) are not, but they are identical to the first 50 msec of the 170 msec CVs. The 170 msec CVs differed in the frequency of F_2_ during the formant transition period with F_0_ and other formant frequencies equal across the three CVs. The 50 msec CVs were created by clipping the 170 msec CVs at the end of the formant transition period (50 msec) using hamming windowing in MATLAB (version R2015a; MathWorks). The first 40 msec of each CV was kept unaltered and >90% reduction in amplitude was applied over the last 10 msec. The resulting 50 msec [ba] [da] and [ga] contained the onset burst and transition period of the original 170 msec CVs without the sustained period. The 40 msec [da] stimulus differed from the 50 and 170 msec CVs in that it contained a longer onset burst and only 5 formants as opposed to the 6 formants in the other CVs (see document, Supplemental Digital Content 1, http://links.lww.com/EANDH/A470, Section 1: Characteristics of CV Stimuli). Polarity of all CVs was reversed using Adobe Audition CC (2015.1 Release, build 8.1.0.162) in order to evoke speech-ABRs using two opposite stimulus polarities as recommended by Skoe and Kraus (2010).

Speech-ABRs in noise were measured using a two-talker-babble masker (used by Song et al. 2011a, b). Two-talker babble was selected over speech spectrum noise as being more representative of real life situations and to ensure that the ABR in noise fell between ceiling (response in quiet) and floor (EEG noise floor). Because two-talker babble contains deep modulations, it degrades the speech-ABR less than the six-talker babble as shown by Song et al. (2011b). Speech-ABRs in two-talker babble have been previously described in response to the 170 msec [da] (e.g., Song et al. 2011b); however, to our knowledge, this is the first study to describe speech-ABRs to the 40 msec [da] in two-talker babble.

#### Recording Parameters

CED Signal software sampling configuration was set to gap-free sweep mode, sample rate of 20,000 Hz, pulses with a resolution of 0.01 msec as the output type, and outputs were set at absolute levels and absolute times. Online artifact rejection was set to reject epochs that included any activity above 20 μV. Stimulus presentation rates were stimulus specific and were set based on the stimulus duration plus an interstimulus interval sufficient to record the response and the baseline (Skoe & Kraus 2010). Because recording time would influence the clinical applicability of the speech-ABR, presentation rates were, therefore, set to reduce recording time to the shortest possible per stimulus (see Table 1 for additional parameters). Two channel vertical electrode montage recording with Cz active, earlobe reference (A1 and A2), and high forehead ground (Fz) was conducted, electrode sites were based on the international 10 to 20 EEG system.

**TABLE 1.**
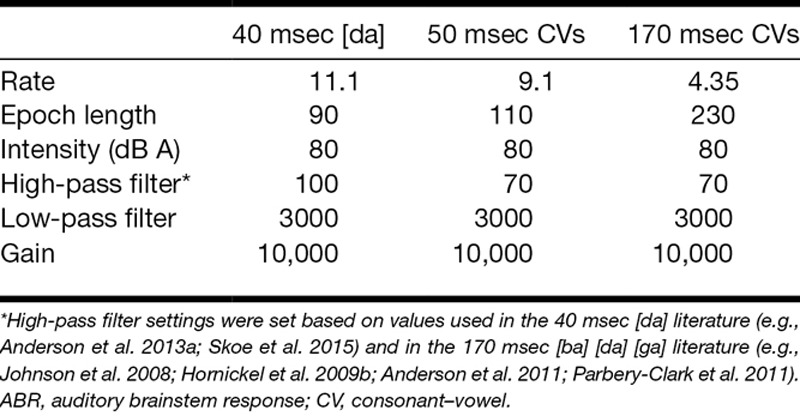
Speech-ABR recording parameters including presentation rate (in stimulus per second), epoch length (recording time window including inter stimulus interval, in milliseconds), sound intensity, high-pass and low-pass filter cutoff frequency (Hz), and amplification (gain)

### Procedure

#### Participant Preparation

Skin at Cz, earlobes (A1 and A2), and high forehead (Fz) was prepared using Nuprep Skin Prep Gel. Ag/AgCI 10 mm disposable disc electrodes were placed on prepared sites with Ten20 Conductive EEG paste and secured with tape at A1, A2, and Fz.

#### Recording Environment

Participants were seated and reclined in a comfortable recliner in a double-wall soundproof booth and instructed to remain relaxed with their eyes closed to reduce myogenic artifacts and eye blinks. Insert earphone was placed in the right ear with the appropriate sized E.A.RLINK foam ear-tip while the left ear remained free. Right ear recording was selected due to the reported right ear advantage for speech-ABR (Hornickel et al. 2009a).

#### Recording Sessions

Speech-ABRs in quiet were collected in response to the 40 msec [da], 50 msec [ba] [da] [ga], and 170 msec [ba] [da] [ga]. Speech-ABRs in two-talker babble at +10 dB signal to noise ratio (SNR) (70 dB A noise and 80 dB A speech) were collected in response to the 40 msec [da], 50 msec [ba] [da] [ga], and only the 170 msec [da]. SNR of +10 dB was set based on speech-ABR literature. Background babble was paused after each block and restarted at the next block to ensure random sections of the babble started with each block. Recordings were completed over 4 to 5 sessions (2 to 3 hours each) across 4 to 5 weeks. Order of the two backgrounds (quiet and noise) and order of CVs and durations were randomized using a Latin square. A total of 12,000 artifact-free epochs were collected per stimulus, 2 blocks of 3000 epochs were collected for each stimulus polarity resulting in a total of 6000 epochs per polarity. Electrode impedances were below 3 kΩ, and impedances between electrodes were balanced and below 1 kΩ. Recording times were documented from the start of the first block until the end of the fourth block per stimulus and background (quiet and noise), including rejected epochs and repeated blocks due to increased EEG artifact. Recording times for speech-ABRs to the 40 msec [da] were slightly shorter than that to the 50 msec CVs. Speech-ABRs to the 170 msec CVs took longest (see document, Supplemental Digital Content 1, http://links.lww.com/EANDH/A470, Section 2: Recording Time Per Stimulus).

### Analyses

#### Processing ABRs

Raw EEG data were processed and analyzed in MATLAB R2015a (MathWorks). The ipsilateral channel (channel 2) was processed for each response. The two blocks of each polarity were averaged separately then low-pass filtered at 2000 Hz as reported in the speech-ABR literature (e.g., Russo et al. 2004; Banai et al. 2009; Anderson et al. 2013b), using the *eegfilt* function of the EEGLAB toolbox (Delorme & Makeig 2004), and converted to microvolts. Filtered responses for each polarity were then averaged together for a final averaged alternating polarity response. Alternating polarity was used to reduce stimulus artifact and cochlear microphonics (Skoe & Kraus 2010). Final responses were then baseline corrected via de-meaning and the first 70 msec were plotted in the time domain to assess peak latencies and peak amplitudes. Time domain analyses were preferred to maintain clinical applicability. Although other analyses techniques are emerging and clinical practice may change in the future, to date, clinical audiologists analyze click and tone burst ABRs in the time domain. Final high-pass filter setting (70 Hz) used for the [ba] [da] [ga] CVs in this study was different than the setting (300 Hz) used by Johnson et al. (2008) and Hornickel et al. (2009b). Johnson et al. and Hornickel et al. reported initially high-pass filtering at 70 Hz, then applying an additional high-pass filter of 300 Hz to emphasize the differences in peak latencies between [ba] [da] and [ga]. However, speech-ABRs recorded for this study were obliterated when high-pass filter was set to 300 Hz; therefore, speech-ABR major and minor peaks identified by Johnson et al. and Hornickel et al. could not be identified in this study (see document, Supplemental Digital Content 1, http://links.lww.com/EANDH/A470, Section 3: Filtering Speech-ABRs to Emphasize Peak Latency Differences Between [ba], [da], and [ga], Section 4: Why Speech-ABRs Contained No Spectral Peaks Above 300 Hz). Thus, all results presented for the [ba] [da] [ga] CVs below were high-pass filtered at 70 Hz.

#### Peak Latency and Amplitude Measurements

To account for the length of the tube of the E.A.RTONE 3A insert earphones, the value of 0.8 msec was subtracted from each peak latency value (Van Campen et al. 1992). Positive peak V and negative peaks A, D, E, F, and O that have been reported in the 40 msec [da] speech-ABR literature (e.g., Skoe & Kraus 2010; Skoe et al. 2015) were visually identified based on published peak latency normative data (Skoe et al. 2015), and their latencies were measured for the 40 msec [da] speech-ABRs. For the 50 and 170 msec CVs, peaks that corresponded to the 40 msec [da] peaks in terms of peak latency and order of occurrence in the response were visually identified, and their latencies were measured. To remain consistent, the same peak nomenclature was used for responses to the 50 and 170 msec CVs. Thus, peak O in response to the 40 and 50 msec CVs is an offset peak, but it is an early FFR peak in response to the 170 msec CVs. Peak (V) to trough (A) amplitudes were measured. For negative peaks D, E, F, and O, the positive peak preceding each peak was used for peak to trough amplitude measurements.

#### Verifying Speech-ABR Quality and Identified Peaks

**Fig. 1.**
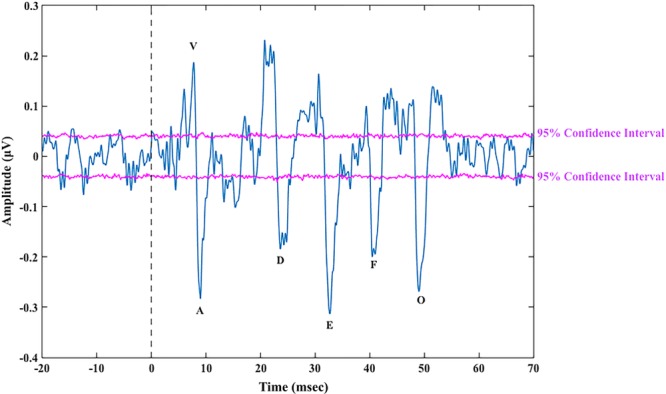
Speech-auditory brainstem response (ABR) with prestimulus baseline to the 40 msec [da] from 1 participant (12,000 epochs) after bootstrap showing all peaks above/below the 95% confidence lines.

Two methods were used to assess quality of responses and ensure 95% confidence that visually identified peaks were above the EEG noise floor. First, the F_SP_ statistic was applied with a criterion of F_SP_ ≥ 3.1 (as described by Don et al. 1984; Elberling & Don 1984). F_SP_ is a measure of the variance in the response over the variance in the background EEG noise, measured by comparing the EEG data within a time region where the response is expected to occur (variance in the response) to the variance of the EEG data at a single time point (variance in the EEG background noise) across averaged epochs (Don et al. 1984; Elberling & Don 1984). Elberling and Don (1984) reported that an F_SP_ of 3.1 equated to 99% confidence that their click-ABRs were present and above the EEG noise floor, and this was measured based on what they termed as a “worst case” (i.e., participants with the highest variance in their background EEG noise). The criterion of F_SP_ ≥ 3.1 set for this study was informed by the work by Don et al. (1984) and Elberling and Don (1984) on click-ABRs as there is no literature on F_SP_ and speech-ABRs. This was applied with the knowledge that there may be individual variability between participants depending on their background EEG noise, differences in filter settings used in this study compared with those used by Don et al. and Elberling and Don, and differences in stimuli (CVs versus clicks). F_SP_ analyses time windows were 5 to 60 msec for responses to 40 msec [da], 8 to 70 msec for responses to both the 50 and 170 msec stimuli. The position of F_SP_ single point was set in the middle of each time window specified above. Speech-ABRs in quiet were considered present if F_SP_ ≥ 3.1. F_SP_ was measured for speech-ABRs in noise; however, because F_SP_ literature only reported results from testing in quiet and there has not been criterion reported for testing in noise, the criterion of 3.1 was not applied to speech-ABRs in noise. Additionally, speech-ABRs in noise have been shown to have lower SNRs compared with speech-ABRs in quiet (Song et al. 2011a; Hornickel et al. 2012); therefore, it is likely that F_SP_ values will also be lower. F_SP_ was measured to no sound recordings, and F_SP_ values were <1.5 (mean = 0.95; SD = 0.25) for all participants, and F_SP_ values of speech-ABRs in noise that did not reach 3.1 were all >1.7 (mean = 2.67; SD = 0.45). Therefore, speech-ABRs in noise were considered present when the F_SP_ at 12,000 epochs was above the participants’ “no sound” F_SP_. Second, the bootstrap method (Efron 1979a, b, 1981)—a method that estimates confidence intervals—was applied (as described by Lv et al. 2007). The bootstrap method does not rely on the variability between participants and can estimate the significance of F_SP_ values for each individual recording. Bootstrap was used to confirm that visually identified peaks were with 95% confidence above the noise floor (Fig. 1), any visually identified peaks that fell outside the 95% confidence lines were considered absent. Both F_SP_ and bootstrap were applied to the 12,000 epochs of speech-ABRs evoked by all stimuli.

#### Determining Number of Epochs Required for a Robust Response

F_SP_ and bootstrap were used to evaluate the number of epochs required to record speech-ABRs with clearly identifiable peaks in response to the 40 and 170 msec [da] in quiet and in noise. Both methods were applied to the averaged alternating polarity speech-ABRs at 15 iterations starting at 800 epochs and increasing by 800 up to 12,000 epochs. The first criterion was the minimum number of epochs required to reach an F_SP_ ≥ 3.1. Once this value was reached, the number of epochs (at or above the number required for F_SP_ ≥ 3.1.) required for all speech-ABR peaks that were detected at 12,000 epochs to be detected with 95% confidence via bootstrap were evaluated for each participant.

#### Degree of FFR Phase Locking

To assess the effect of background noise on the FFR, intertrial phase clustering (degree of phase locking) to F_0_ of the stimulus was implemented on the FFR period (70 to 190 msec) of the raw EEG responses to the 170 msec [da] in quiet and in noise using the method recommended by Cohen (2014). Intertrial phase clustering is the length of the average vector measured by extracting the phase angle for a specific frequency (F_0_ in this study) at each time point from each epoch and calculating the average vector length from the distribution of phase angles in a polar plane, resulting in a value between 0 and 1. Values closer to 1 indicate similar phase angles and thus a higher degree of phase locking, and values closer to 0 indicate minimal degree of phase locking at a particular time point (Cohen 2014). Phase locking analyses focused on F_0_ as it was the most robust component present in speech-ABRs of all participants.

#### Statistical Analyses

##### Effect of Background

The effect of background (quiet versus noise) on peak latencies and peak amplitudes of speech-ABR peaks (V, A, D, E, F, O) was evaluated through fitting linear mixed models (LMM) in R (R Core Team 2016) using *lemer* of the *lme4* package (Bates et al. 2015) and *lmerTest* (Kuznetsova et al. 2016). LMMs allow for unbalanced designs and account for missing data points (e.g., missing peaks in some participants). Two LMMs were fit to the data: (1) latency model was set up with “background” (quiet and noise), “duration” (40, 50, and 170 msec), “peak” (V, A, D, E, F, O), and interaction between “duration” and “peak” as fixed effects and “participants” as random effects, (2) amplitude model was set up with “background,” “duration,” “peak,” interaction between “background” and “peak”, interaction between “background” and “duration,” and interaction between “duration” and “peak” as fixed effects and “participants” as random effects. LMMs were built by conducting a likelihood ratio test to compare an LMM with a fixed effect to a LMM without the fixed effect as described by Winter (2013). Fixed effects that had a significant effect on the LMM (*p* < 0.05) plus LMMs that resulted in a better fit to the data in terms of lower Akaike information criterion were finally selected. More complex LMMs with random intercepts were attempted; however, these models did not converge. The LMM without “CV” ([ba], [da], [ga]) as a fixed effect was a better fit to the data; therefore, “CV” was dropped as a fixed effect from both latency and amplitude models.

Next, the effect of background on the FFR period of the speech-ABR to the 170 msec [da] was evaluated by conducting a 2-tailed paired sample *t* test using R on the Fisher-*Z* transformed maximum degree of phase locking to the fundamental frequency (F_0_) in quiet versus in noise.

##### Effect of Stimulus Duration

The effect of stimulus duration on peak latencies and peak amplitudes of speech-ABR peaks (V, A, D, E, F, O) was evaluated via conducting two LMMs that were the best fit to the data: (1) latency model was set up with “background” (quiet and noise), “duration” (50 and 170 msec), “peak” (V, A, D, E, F, O), and interaction between “duration” and “peak” as fixed effects and “participants” as random effects, (2) amplitude model was set up with “background”, “duration”, “peak”, and interaction between “duration” and “peak” as fixed effects and “participants” as random effects. The duration comparison was restricted to the 50 and 170 msec CVs and the 40 msec [da] was excluded due to the spectral differences in the stimulus that may influence results.

##### Effect of CV

To evaluate the effect of CV on peak latencies, a simpler LMM latency model was built using only speech-ABRs in quiet to 50 and 170 msec [ba] [da] [ga], with “CV” and interaction between “peak” and “CV” as fixed effects and “participants” as random effects.

All post hoc pairwise comparisons were conducted using the *lsmeans* (Lenth 2016) R package. Bonferroni correction was applied to all *p* values to correct for multiple comparisons. Criterion for significance was considered as *p* < 0.01.

## RESULTS

### Detected Peaks

Most peaks were detected with 95% confidence via bootstrap in speech-ABRs of all participants in quiet, with more peaks missing in speech-ABRs in noise than in quiet (see document, Supplemental Digital Content 2, http://links.lww.com/EANDH/A471, Section 1: Detection of Speech-ABR Peaks). The most commonly missing peak was V in noise in speech-ABRs to all stimuli excluding the 40 msec [da], where *F* was the peak most commonly missing in speech-ABRs in noise.

### Effect of Background

#### Peak Latencies

**Fig. 2.**
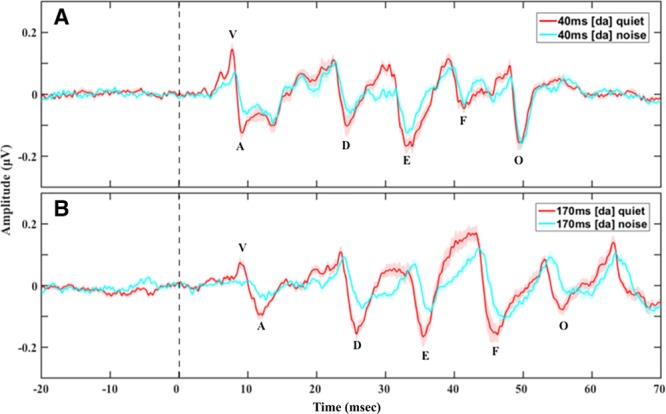
Grand average speech-auditory brainstem responses (ABRs) with prestimulus baseline in quiet and in noise to the (A) 40 msec [da] and (B) 170 msec [da] showing longer peak latencies and smaller peak amplitudes in noise compared with in quiet across the two [da] durations. Shade in all panels represents 1 SE.

**Fig. 3.**
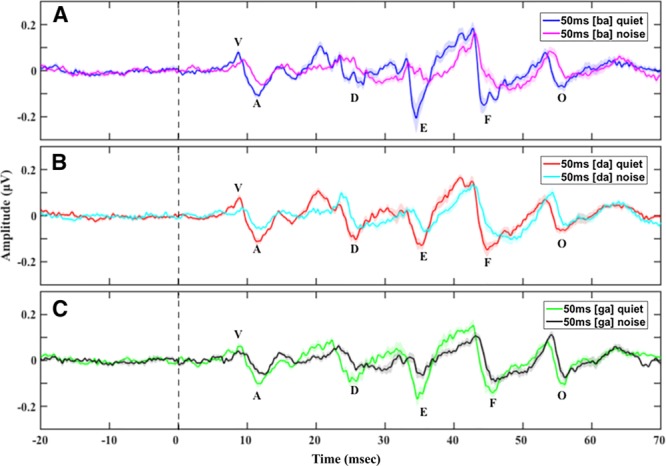
Grand average speech-auditory brainstem responses (ABRs) with prestimulus baseline in quiet and in noise to the (A) 50 msec [ba], (B) 50 msec [da], and (C) 50 msec [ga] showing longer peak latencies and smaller peak amplitudes in noise compared with in quiet across the three consonant–vowels (CVs). Shade in all panels represents 1 SE.

Background had a significant effect on speech-ABR peak latencies (*b* = 0.91; *t*(796.10) = 9.42; *p* < 0.01; Figs. 2 and 3). Peak latencies in noise were longer than peak latencies in quiet for all stimulus durations. Post hoc pairwise comparisons to investigate the effect of “background” on specific peak latencies revealed that latencies of all peaks were significantly longer (*p* < 0.01) in speech-ABRs in noise compared with in quiet regardless of stimulus duration (see document, Supplemental Digital Content 2, http://links.lww.com/EANDH/A471, Section 2: Speech-ABR Mean (SD) Peak Latencies and Amplitudes, Section 3: Effects of Background on Speech-ABRs – Post Hoc Pairwise Comparison Results).

#### Peak Amplitudes

Peak amplitudes for speech-ABRs in noise were significantly smaller than peak amplitudes in quiet (*b* = −0.12; *t*(687.00) = −6.24; *p* < 0.01; Figs. 2 and 3). There was a significant interaction between “background” and “peak” (χ^2^(1) = 30.09; *p* < 0.01) as revealed by the likelihood ratio test. Post hoc pairwise comparisons to investigate the effect of “background” on specific peak amplitudes revealed that all speech-ABR peaks had significantly smaller amplitudes (*p* < 0.01) in noise compared with in quiet regardless of stimulus duration, excluding peak O that had a similar amplitude in quiet and in noise (see document, Supplemental Digital Content 2, http://links.lww.com/EANDH/A471, Section 2: Speech-ABR Mean (SD) Peak Latencies and Amplitudes, Section 3: Effects of Background on Speech-ABRs – Post Hoc Pairwise Comparison Results).

#### Degree of Phase Locking

**Fig. 4.**
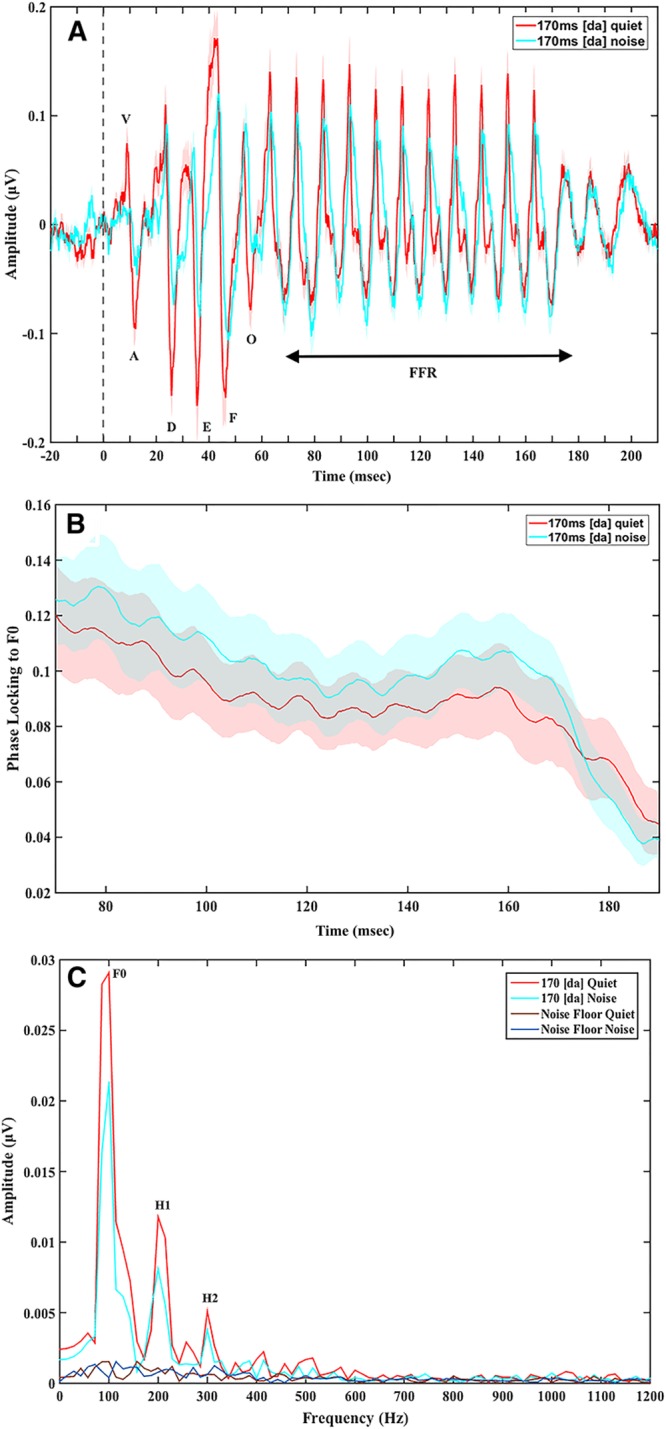
Grand average speech-auditory brainstem responses (ABRs) in quiet and in noise to the 170 msec [da]: (A) time domain waveforms with prestimulus baseline, showing greater effect of noise on onset and transition peaks (0 to 70 msec) than on the later frequency following response (FFR) period (70 to 190 msec), (B) FFR degree of phase locking to F_0_, showing a nonsignificant trend for higher degree of phase locking to F_0_ in noise compared with in quiet. Shade in all panels represents 1 SE. C, Spectrum (fast Fourier transform) of the onset and transition period (0 to 70 msec) showing the greater effect of noise on F_0_ in the first 70 msec or the response.

Greater FFR degree of phase locking to F_0_ was found in speech-ABRs in noise relative to in quiet (Fig. 4), though this difference was not significant (*t*(21.97) = −0.29; *p* = 0.78).

### Effect of Stimulus Duration

#### Peak Latencies

**Fig. 5.**
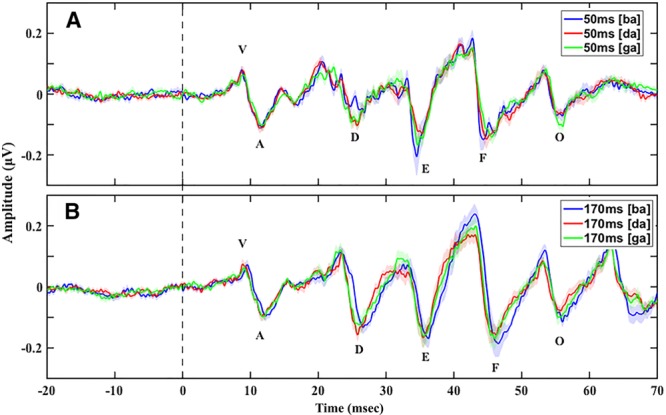
Grand average speech-auditory brainstem responses (ABRs) with prestimulus baseline in quiet to the (A) 50 msec [ba] [da] [ga] and (B) 170 msec [ba] [da] [ga] showing no differences in peak latencies and amplitudes between the two stimulus durations and no differences in peak latencies between the three consonant–vowels (CVs) across the two stimulus durations. While responses to 170 msec [ba] seem to have longer peak latencies, this was not significant. Shade in all panels represents 1 SE.

Stimulus duration (50 versus 170 msec) did not have a significant effect on speech-ABR peak latencies (*b* = 0.74; *t*(667) = −2.815; *p* = 0.09; Fig. 5).

#### Peak Amplitudes

Peak amplitudes for speech-ABRs to the 50 msec CVs were significantly smaller than to 170 msec CVs (*b* = −0.07; *t*(578) = −3.83; *p* < 0.01; Fig. 5). There was a significant interaction between “duration” and “peak” (χ^2^(1) = 18.46; *p* < 0.01) as revealed by the likelihood ratio test. Post hoc pairwise comparisons to investigate the effect of “duration” and the interaction between “duration” and “peak” on specific peak amplitudes revealed that only peak D amplitude was significantly smaller (*p* < 0.01) in speech-ABRs to the 50 msec CVs compared with the 170 msec CVs both in quiet and in background noise.

### Effect of CV

CV had no effect on peak latencies ([da]: *b* = −0.04; *t*(396) = −0.10; *p* = 0.92 and [ga]: *b* = −0.01; *t*(396) = −0.10; *p* = 0.99; Fig. 5); however, there was a significant interaction between “peak” and “CV” (χ^2^(1) = 2201.90; *p* < 0.01) as revealed by the likelihood ratio test. Post hoc pairwise comparison to investigate this interaction revealed no significant effect of “CV” on peak latencies when comparison was on the same peak and a different CV (e.g., peak D and CV [ba] versus peak D and CV [ga]). Some authors (e.g., Skoe et al. 2011) have suggested using a “cross-phasogram” approach to explore how the phase of components in speech-ABRs to different CVs may vary. This approach uses the cross-power spectral density between the responses to 2 CVs to calculate phase differences between the responses over time and frequency. Use of this approach for analyses of speech-ABRs from this study was not appropriate due to the following: (1) phase measurements are very sensitive to background noise, and this generally increases when responses are combined; (2) the analyses will include frequencies that are not harmonics of the fundamental frequency in the response and hence phase would be calculated at frequencies where no response would be expected, which introduces difficulty in interpretation; and (3) the robustness and efficacy of the cross-phasogram has not yet been well tested.

### Number of Epochs

The numbers of epochs required to reach F_SP_ ≥ 3.1 varied among participants, which may reflect variations in the background EEG noise characteristics between participants. In general, speech-ABRs in quiet required a smaller number of epochs to reach F_SP_ ≥ 3.1 than speech-ABRs in noise to both 40 and 170 msec [da]. In 2 participants, speech-ABRs in noise to the 170 msec [da] did not reach F_SP_ ≥ 3.1 (F_SP_ = 2.96; F_SP_ = 2.95) at 12,000 epochs; however, their speech-ABR peaks were detected with 95% confidence via bootstrap. Although criterion of F_SP_ ≥ 3.1 indicates that response is present, it does not imply that all peaks can be detected, as some participants required more epochs for all peaks to be detected with 95% confidence via bootstrap than to reach F_SP_ ≥ 3.1. Specifically, in speech-ABRs to the 40 msec [da], 3 participants required 800 more epochs in order for all peaks to be detected in their speech-ABRs in quiet, and 5 participants required a larger number of epochs (1 required 800, 2 required 1600, and 2 required 4000 more epochs) for all peaks to be detected in their speech-ABRs in noise. In speech-ABRs to the 170 msec [da], 7 participants required larger number of epochs (1 required 1600, 2 required 2400, 2 required 3200, 1 required 4000, and 1 required 4800 more epochs) for all peaks to be detected in their speech-ABRs in quiet, and 5 participants required larger number of epochs (2 required 800, 1 required 2400, and 2 required 4800 more epochs) for all peaks to be detected in their speech-ABRs in noise (see document, Supplemental Digital Content 2, http://links.lww.com/EANDH/A471, Section 4: Bootstrap Results and Examples). Average F_SP_ values where all peaks were detected with 95% confidence via bootstrap for speech-ABRs in quiet were 4.17 (SD = 0.91; range: 3.16 to 6.17) for the 40 msec [da] and 6.94 (SD = 3.65; range: 3.25 to 12.42) for the 170 msec [da] and for speech-ABRs in noise were 4.24 (SD = 1.15; range: 3.14 to 6.16) for the 40 msec [da] and 4.30 (SD = 1.82; range: 3.21 to 8.86) for the 170 msec [da] (see document, Supplemental Digital Content 2, http://links.lww.com/EANDH/A471, Section 5: Fsp Values and Number of Epochs).

## DISCUSSION

The aims of this study were to evaluate the effects of background, stimulus duration, and CV on speech-ABRs. Hence, the differences in speech-ABRs recorded to 3 CVs of short duration (40 and 50 msec) and long duration (170 msec) presented in two backgrounds (quiet and noise) were assessed. This was done to establish whether shorter CVs, which would be more clinically applicable due to shorter test-time (1) can be reliably used for speech-ABRs in noise, (2) evoke robust ABRs comparable to ABRs evoked by long CVs, and (3) can be used to assess discrimination between CVs. A secondary aim of this study was to evaluate the number of epochs required to achieve a speech-ABR with clearly identifiable peaks. It is worth noting that results from this study apply to recording speech-ABRs at 80 dB A, and response quality may be reduced if lower presentation levels are to be used.

### Speech-ABR in Background Noise

Speech-ABR peak latencies were longer and amplitudes were smaller in noise than in quiet across the three durations and the three CVs, excluding amplitude of peak O that was not affected by background noise. Additionally, there were more speech-ABR peaks missing in noise than in quiet. These results are in general agreement with published results on speech-ABRs in noise for the 40 and 170 msec [da] (Russo et al. 2004; Parbery-Clark et al. 2011; Song et al. 2011a). Results are also in agreement with published results on click-ABRs in noise that found a delay in click-ABR peak V (analogous to speech-ABR peak V) latency when background noise was added (e.g., Burkard & Sims 2002; Mehraei et al. 2016). However, Parbery-Clark et al. (2011) reported that only onset peaks had reduced amplitudes in noise compared with in quiet, with longer latencies of both onset and transition peaks in noise and Song et al. (2011a) reported that only onset peaks V and A had delayed latencies with no difference in latencies of transition peaks between quiet and noise. Parbery-Clark et al. recorded speech-ABRs binaurally to the 170 ms [da], binaural presentation is known to result in more robust responses (Skoe & Kraus 2010), which may explain the lack of change in amplitudes in transition peaks found by Parbery-Clark et al. While there were no notable methodological differences between this study and the study by Song et al. The reasons behind our longer peak latencies and smaller peak amplitudes in noise compared with in quiet are unclear. Burkard and Sims (2002) attributed click-ABR peak V latency delay to neural desynchronization. Mehraei et al. also stipulated that neural desynchronization resulted in delayed click-ABR peak V latency, more specifically that low spontaneous rate auditory nerve fibers that are slower to fire are the main contributors to ABRs in noise, while high spontaneous rate auditory nerve fibers contribute less because they are more affected by background noise. Another reason may be that the addition of background noise may result in a shift in cochlear-place of the response, as it has been shown that speech-ABRs in quiet that originated from a lower-frequency cochlear region had longer peak latencies and smaller peak amplitudes (Nuttall et al. 2015). Furthermore, the lack of difference in peak O amplitudes in noise compared with in quiet may be a result of compensation that occurs in the brainstem pathway as stipulated by Russo et al. (2004). In terms of the effect of background on the FFRs degree of phase locking to F_0_ of the stimulus, we found no significant difference between speech-ABR FFRs in quiet and in noise. This lack of effect of background noise on F_0_ is consistent with earlier reports (Li & Jeng 2011; Song et al. 2011b; Smalt et al. 2012). Li and Jeng (2011) also found that F_0_ of the FFR did not decrease in amplitude with positive dB SNR levels; it was only affected at 0 dB SNR and negative dB SNR levels. While AlOsman et al. (2016) and Prévost et al. (2013) found an enhancement in FFR F_0_ in background noise compared with in quiet, AlOsman et al. stipulated that this enhancement was modulated by top-down processing to improve speech understanding in background noise, while Prévost et al. attributed this enhancement to the phase locking to the stimulus envelope of auditory nerve fibers that are further away from the characteristic frequency of F_0_, in order to compensate for the effect of background noise. Involvement of the auditory cortex in the FFR has been shown by Coffey et al. (2016) in their FFR and magnetoencephalography study where auditory cortical activation at F_0_ of the stimulus was found in normal-hearing adults. This supports top-down modulation of the FFR and may explain the lack of effect of background noise on phase locking to F_0_ that was found in this study. However, a significant effect of background on peak latencies and amplitudes occurring in the first 60 to 70 msec of the speech-ABR was found. Physiological reasons behind these effects remain unclear as physiological mechanisms related to speech perception in noise within the peripheral auditory system and the brainstem are still not fully resolved in the literature. Further investigation of these physiological mechanisms is needed. Nonetheless, the effect of background noise on speech-ABR peak latencies and amplitudes was similar across the three CV durations in this study, and the FFR period (70 to 190 msec) of the speech-ABR to the longer duration stimulus was not affected by background noise at +10 dB SNR. These results suggest that peaks occurring in the first 60 to 70 msec of speech-ABRs to all stimulus durations are equally influenced by noise with the FFR period to the longer stimulus durations not being affected by noise. The FFR period would likely require higher background noise levels to be affected, which would require higher presentation levels that may be uncomfortably loud to some individuals as was revealed during the pilot for this study.

### Speech-ABRs and Stimulus Duration

Speech-ABR peak latencies and peak amplitudes were similar across the 50 and 170 msec CVs. Although faster presentation rates have been reported to delay onset peak latencies (Krizman et al. 2010), this was not the case in this study. Peak latencies of speech-ABRs to 170 msec CVs (presented at 4.35 stimuli per second) were similar to those in response to the 50 msec CVs (presented at 9.1 stimuli per second). These results suggest that stimulus duration does not affect speech-ABR peak latencies or peak amplitudes when shorter and longer versions of the same stimuli are used, and all speech-ABR peaks are identifiable across the two durations (50 and 170 msec). Therefore, any stimulus duration may be used to record speech-ABRs, assuming stimulus-specific normative data is established.

### Speech-ABR and CV Discrimination

Speech-ABR peak latencies to the three CVs ([ba] [da] [ga]) were similar across the three CVs and two durations (50 and 170 msec) in quiet. These results are at odds with results from Johnson et al. (2008) and Hornickel et al. (2009b) who found overall earlier peak latencies for the 170 msec [ga] compared with the 170 msec [da] and [ba], and overall later peak latencies for the 170 msec [ba] compared with the 170 msec [da] and [ga]. Speech-ABR high-pass filter cutoff frequency used by Johnson et al. and Hornickel et al. was 300 Hz. High-pass filtering speech-ABRs from this study at such a high frequency resulted in complete loss of the response, thus the major and minor peaks that were identified by Johnson et al. and Hornickel et al. could not be identified in speech-ABRs from this study. The reasons behind differences between speech-ABRs recorded in this study and those recorded by Johnson et al. and Hornickel et al. are unclear. Speech-ABRs from this study contained little to no spectral peaks above 300 Hz, which rendered high-pass filtering at 300 Hz redundant. Additionally, the spectra of speech-ABRs from this study were a very good match to the predicted spectra obtained from analyzing the half-wave rectified acoustic CV stimuli (these same CVs were used in Johnson et al. 2008; Hornickel et al. 2009b) that also contained no clear spectral peaks above 300 Hz. Therefore, it is unclear what is driving the high-frequency content in speech-ABRs reported by Johnson et al. and Hornickel et al. Also, the three CVs only differ in the vowel formant frequency of F_2_, which is above the reported maximum frequency (approximately 1034 Hz) that the brainstem is able to phase-lock to (Liu et al. 2006). Results from this study suggest that the speech-ABR may not be a useful tool to assess auditory discrimination between these specific CVs that differ in F_2_ frequency regardless of CV duration.

### Number of Epochs

Number of epochs required for recording speech-ABRs with clearly identifiable peaks varied between participants; they were as low as 1600 in quiet and 2400 in noise and as high as 6400 in quiet and 12,000 in noise. Number of epochs required for speech-ABRs in noise was generally larger than in quiet to both the 40 and 170 msec [da]. Speech-ABRs to the 40 msec [da] in quiet and in noise of most participants (total = 8) required a smaller number of epochs to reach a combination of F_SP_ ≥ 3.1 and peaks detected with 95% confidence via bootstrap than to the 170 msec [da]. Plus, speech-ABRs in noise to 170 msec [da] did not reach F_SP_ ≥ 3.1 at 12,000 epochs in 2 participants. Fewer epochs to achieve speech-ABRs with clearly identifiable peaks in response to the 40 msec [da] would encourage its’ clinical application as fewer epochs combined with the shorter stimulus duration would require shorter testing sessions than longer duration stimuli combined with more epochs.

Due to this variability in the number of epochs, implementing an automated method such as the combination of F_SP_ and bootstrap during speech-ABR recording would assist clinicians and researchers in identifying the number of epochs required for a particular individual, in addition to being confident that responses are present and that identified/detected peaks are above the background EEG noise. Applying such methods online while recording would save time in those that require fewer epochs and would increase the likelihood of response detection in those that require a larger number of epochs. Bootstrap approaches have the advantage over the F_SP_ in that they are less influenced by variability in recordings between participants; however, they are more computationally complex to implement. Therefore, applying F_SP_ online during recording until a certain criterion is reached (e.g., 3.1), then applying bootstrap online after this criterion is reached, would likely be more feasible. However, more work is needed to determine the appropriate F_SP_ values that correspond to 99% confidence response presence in speech-ABRs in quiet and in noise and to determine the most sensitive measure for detection of speech-ABRs.

## CONCLUSIONS

This is the first study that systematically investigated the clinical feasibility of speech-ABRs as an objective audiological measure. The speech-ABR was evaluated in terms of stimulus duration, background noise, and number of epochs within the same participants. The results show that the 40 msec [da] in quiet and in noise is the most appropriate stimuli for the clinical implementation of the speech-ABR to evaluate speech detection and speech-ABRs in noise, based on the following:

1. The influence of background on peak latencies and amplitudes is similar across stimuli regardless of duration, with no effect of background on the FFR in speech-ABRs to longer duration stimuli.
2. The lack of peak latency differences in speech-ABRs between the three CVs (regardless of duration) suggests that the speech-ABR may not be an appropriate tool to assess auditory discrimination of the CV stimuli used in this study.
3. Fewer epochs are required to record speech-ABRs with clearly identifiable peaks to the 40 msec [da], this combined with the short stimulus duration leads to shorter session times.

## FUTURE DIRECTIONS

Several features of the speech signal may be recorded via speech-ABRs, these include (1) sound onset; (2) frequency transitions; (3) formant structure; and (4) F_0_ (Kraus & Nicol 2005; Abrams & Kraus 2015). Such features cannot be measured using current clinical click and tone burst ABRs. The speech-ABR could, therefore, be a valuable clinical tool in the assessment of subcortical encoding of speech in quiet and in background noise. In this study, four issues related to the clinical feasibility of speech-ABRs were addressed: (1) stimulus duration, (2) background (quiet versus noise), (3) CV, and (4) number of epochs. Results from this study add to existing speech-ABR literature and are a step forward toward the development of clinical protocols for speech-ABRs. More specifically for the development of clinical protocols for speech-ABRs as a measure of subcortical encoding of speech and of speech-in-noise performance. However, ample work is still needed before speech-ABRs can be introduced to clinical practice. For example, before the clinical application of speech-ABRs as a measure of speech-in-noise performance, stimulus-specific normative data on speech-ABRs in quiet versus in noise in normal-hearing individuals and in clinical populations (e.g., individuals with hearing loss) are necessary, such studies should ideally include criteria for what is considered a normal change in speech-ABRs with the addition of background noise and what would indicate degradation in speech-in-noise performance. Further investigation is also needed using CVs different than those used in this study to evaluate the speech-ABRs usability as a measure of discrimination of speech sounds before its clinical application for this purpose. Finally, there is a need to establish a sensitive clinically feasible measure for speech-ABR detection and confirmation of response presence (e.g., appropriate F_SP_ values that correspond to 99% confidence response presence combined with bootstrap).

## ACKNOWLEDGMENTS

The authors thank Dr Timothy Wilding, Dr Emanuele Perugia, and Dr Frederic Marmel at the Manchester Centre for Audiology and Deafness, School of Health Sciences, Faculty of Biology, Medicine and Health, University of Manchester, Manchester Academic Health Science Centre for their help in writing the MATLAB code for some of the data processing. The authors also thank the Auditory Neuroscience Laboratory, Department of Communication Sciences, Northwestern University, Evanston, IL, USA for the provision of stimuli (consonant vowels and background babble) used in this study. G.B. designed and performed the experiment, analyzed the data, and wrote the paper; A.L. was involved in experiment design and interpretation of results; S.L.B. and G.P. were involved in data processing, MATLAB coding, and reviewed results; M.O. was involved in study setup and reviewed results; K.K. was involved in experiment design, data analyses, and interpretation of results. All authors discussed results and commented on the manuscript at all stages.

This research was funded by the Saudi Arabian Ministry of Education and King Fahad Medical City (to G.B.) and by the Engineering and Physical Sciences Research Council grant EP/M026728/1 (to K.K. and S.L.B.). This study was supported by the NIHR Manchester Biomedical Research Centre.

Portions of this article were previously presented at the XXV International Evoked Response Audiometry Study Group Biennial Symposium, Warsaw, Poland, May 22, 2017; at the 40th MidWinter Meeting of the Association for Research in Otolaryngology, Baltimore, MD, USA, February 12, 2017; and at the Basic Auditory Science Meeting, Cambridge, United Kingdom, September 5, 2016.

Raw EEG data (speech-ABRs) for this study may be accessed at (https://zenodo.org/record/1284997#.W2BueS2Q0ch).

## Supplementary Material
