# Supplementary material for: Speech Auditory Brainstem Responses: Effects of Background, Stimulus Duration, Consonant–Vowel, and Number of Epochs
Source: Ear Hear. 2019 Apr 26;40(3):659–70. doi: 10.1097/AUD.0000000000000648 (PMC6493675; doi:10.1097/AUD.0000000000000648)
Supplement: Supplementary file 1 [file aud-40-659-s001.pdf]

Supplemental Digital Content 1

Speech Auditory Brainstem Responses: Effects of Background, Stimulus Duration, Consonant-Vowel, and Number of Epochs

Section 1: Characteristics of CV Stimuli

Table 1. Formant Frequency Components ($F_0 - F_6$, in Hz) of CV Stimuli used to Record Speech-ABRs.

↑ = *frequency rises during vowel formant transition.*

↓ = *frequency falls during vowel formant transition.*

	**40ms**	**50ms**			**170ms**		
	**[da]**	**[ba]**	**[da]**	**[ga]**	**[ba]**	**[da]**	**[ga]**
F_0	103 ↑ 125	100			100		
F_1	220 ↑ 720	400 ↑ 720			400 ↑ 720		
F_2	1700 ↓ 1240	900 ↑ 1240	1700 ↓ 1240	2480 ↓ 1240	900 ↑ 1240	1700 ↓ 1240	2480 ↓ 1240
					Frequency transition during the first 50ms		
F_3	2580 ↓ 2500	2580 ↓ 2500			2580 ↓ 2500		
F_4	3600	3300			3300		
F_5	4500	3750			3750		
F_6	NA	4900			4900		

40ms [da] from (Banai et al. 2009)

170ms [ba] [da] [ga] from (Hornickel et al. 2009)

Time Domain Waveforms of Stimuli

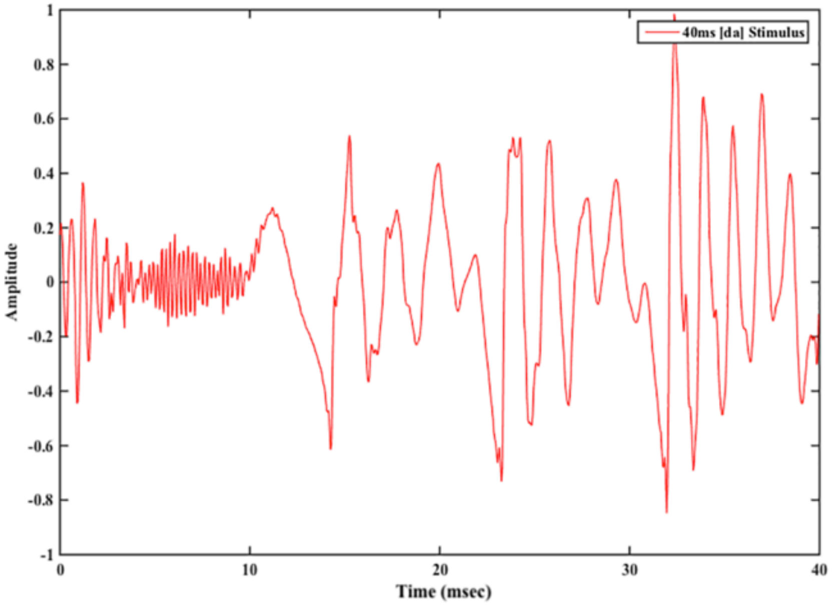

Fig. 1. Time domain waveform of a single polarity 40ms [da] stimulus

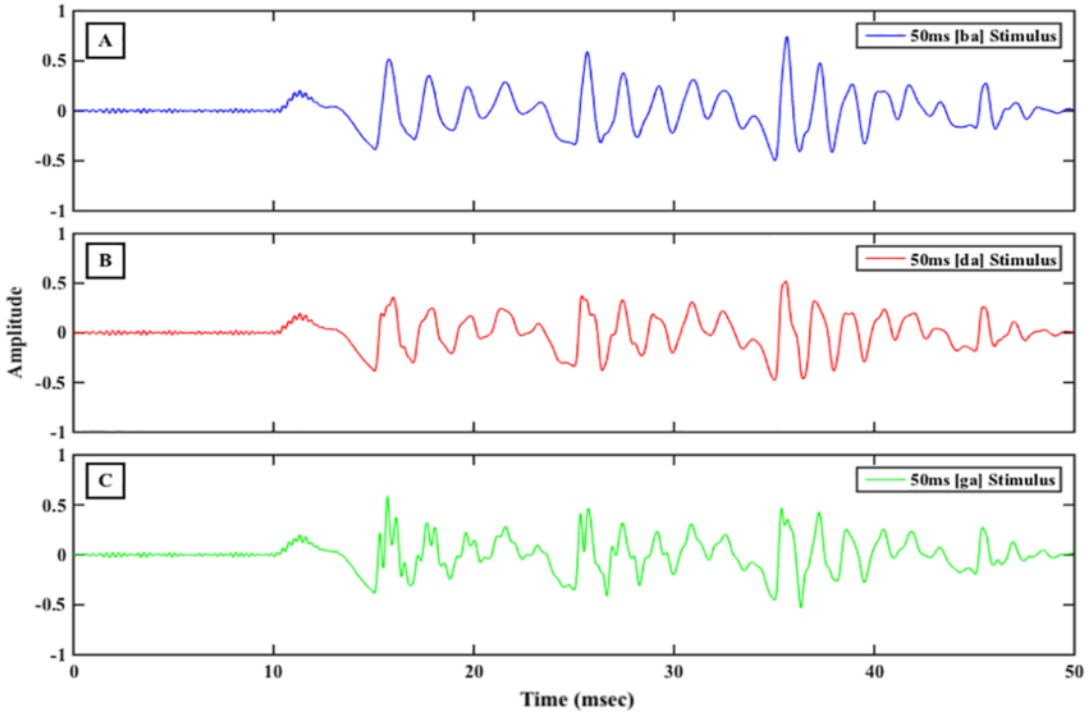

Fig. 2. Time domain waveforms of a single polarity: (A) 50ms [ba] stimulus, (B) 50ms [da] stimulus, (C) 50ms [ga] stimulus

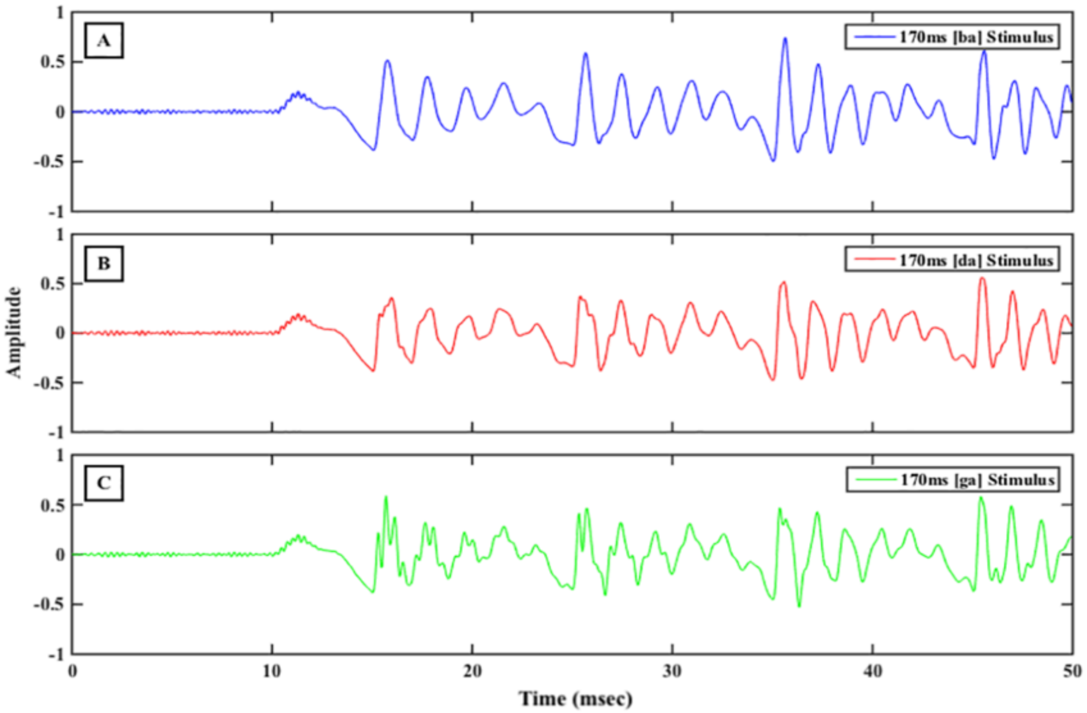

Fig. 3. Time domain waveforms of the transition period (first 50ms) of a single polarity: (A) 170ms [ba] stimulus, (B) 170ms [da] stimulus, (C) 170ms [ga] stimulus

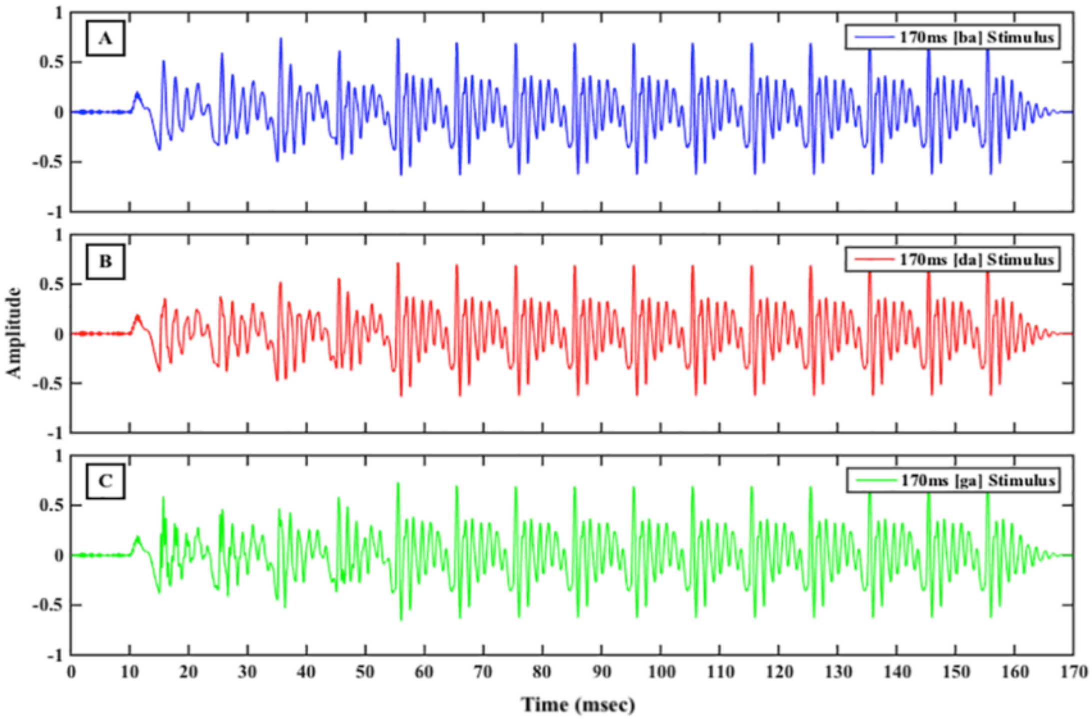

Fig. 4. Time domain waveforms of a single polarity: (A) 170ms [ba] stimulus, (B) 170ms [da] stimulus, (C) 170ms [ga] stimulus

Spectrum of Stimuli

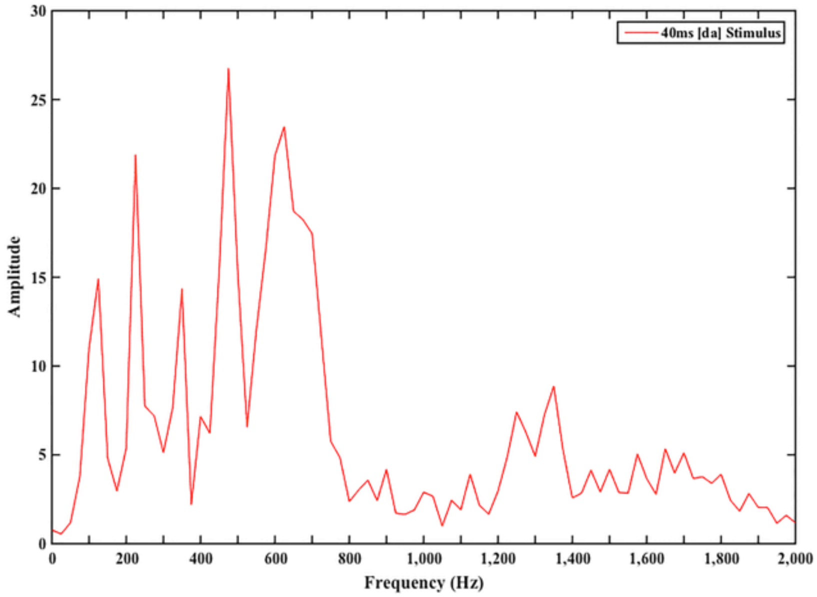

Fig. 5. Spectrum (FFT of the full stimulus) of a single polarity 40ms [da] stimulus

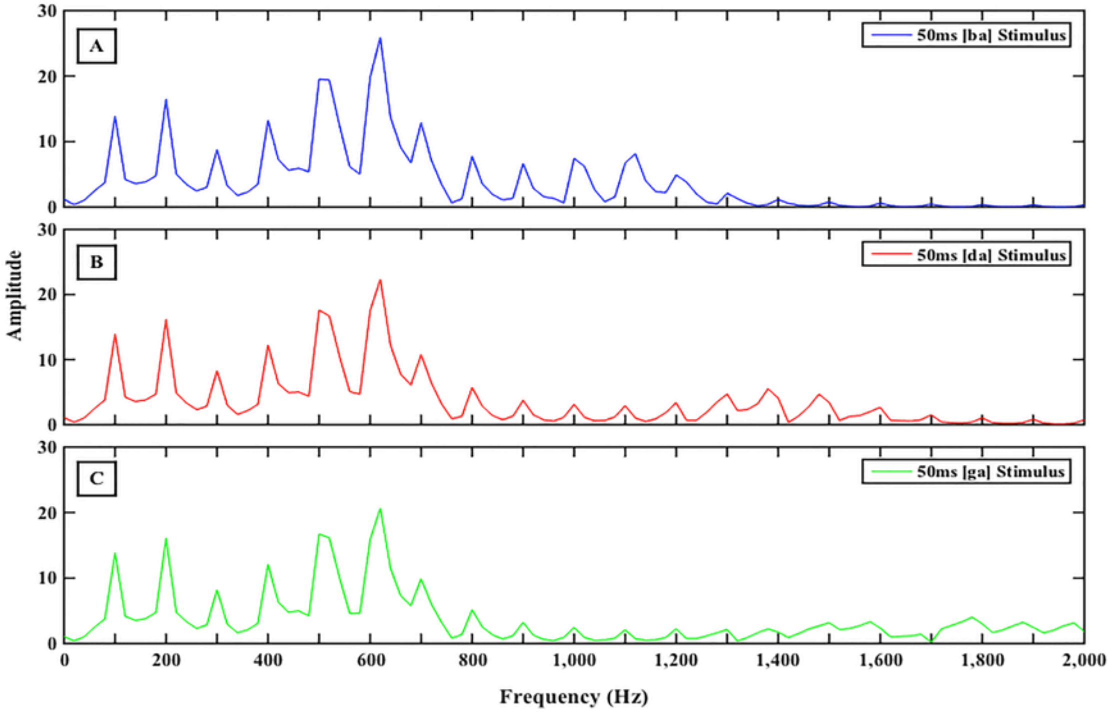

Fig. 6. Spectrum (FFT of the full stimulus) of a single polarity: (A) 50ms [ba] stimulus, (B) 50ms [da] stimulus, (C) 50ms [ga] stimulus

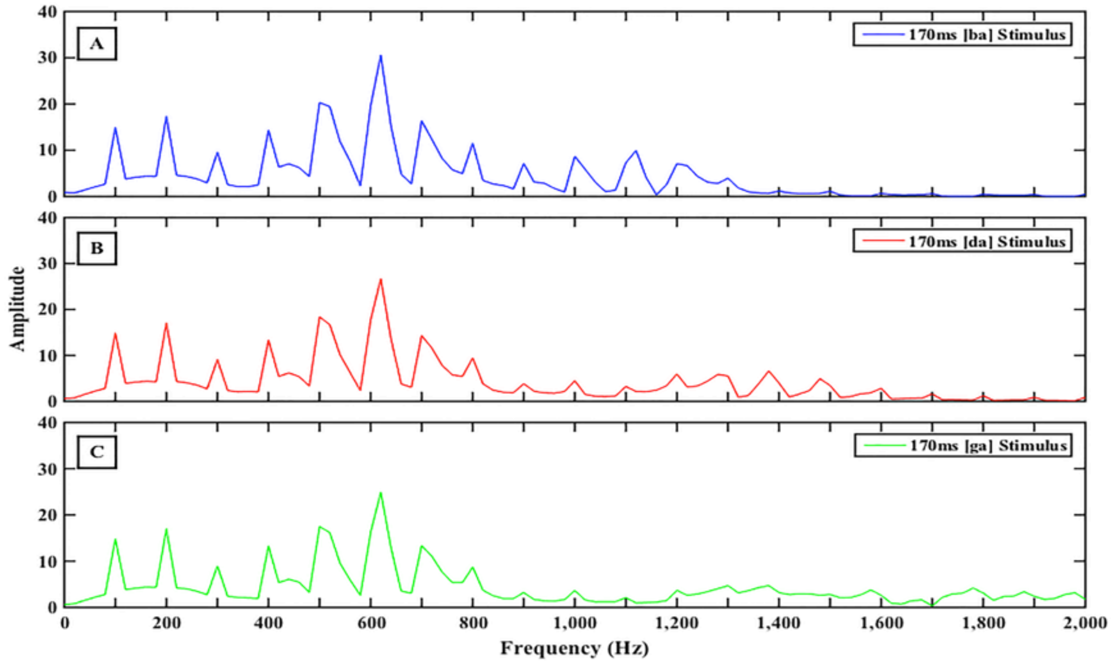

Fig. 7. Spectrum (FFT of the first 50ms of the stimulus) of the transition period (first 50ms) of a single polarity: (A) 170ms [ba] stimulus, (B) 170ms [da] stimulus, (C) 170ms [ga] stimulus

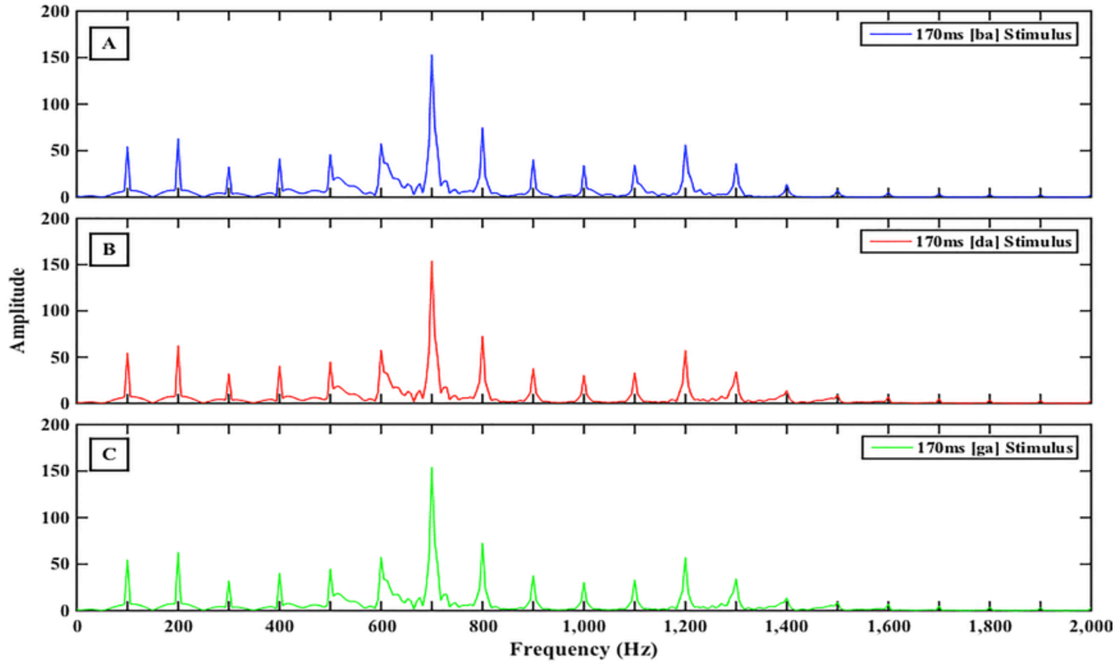

Fig. 8. Spectrum (FFT of the full stimulus) of a single polarity: (A) 170ms [ba] stimulus, (B) 170ms [da] stimulus, (C) 170ms [ga] stimulus

Section 2: Recording Time Per Stimulus

Table 2. Mean, SD, and Range of Recording Times (in minutes) required for completing 4 Speech-ABR blocks (i.e. 12,000 epochs) per stimulus, across durations (40ms, 50ms, 170ms), and in each background (quiet and noise).

Shaded cells indicate that stimulus was not tested in noise.

		Quiet			Noise		
		**Mean**	**SD**	**Range**	**Mean**	**SD**	**Range**
**40ms**	**[da]**	23.17	5.22	20 – 31	21.75	3.19	19 – 25
**50ms**	**[ba]**	27.08	3.94	24 – 36	30.17	7.52	25 – 49
	**[da]**	27.75	6.45	24 – 45	28.17	5.64	23 – 45
	**[ga]**	29.17	5.75	25 – 40	30.33	7.45	25 – 47
**170ms**	**[ba]**	56.08	15.92	48 – 106			
	**[da]**	54.50	8.92	48 – 81	56.00	7.48	49 – 71
	**[ga]**	53.92	7.04	49 – 73			

Section 3: Filtering Speech-ABRs to Emphasize Peak Latency Differences Between [ba], [da], and [ga]

Johnson et al. 2008 and Hornickel et al. 2009 reported first band-pass filtering speech-ABRs to each stimulus polarity from 70 – 2000 Hz, then adding speech-ABRs to the 2 polarities. Following filtering and adding speech-ABRs, an additional high-pass filter of 300 Hz was applied to the added speech-ABRs.

In speech-ABRs that were recorded in this study, applying the additional high-pass filter to the added responses resulted in a drastic decrease in speech-ABR amplitudes with no clearly defined peaks (Figs.9, 10, 11, 12). A spectrum of speech-ABR onset and transition periods to these 3 stimuli shows that speech-ABRs from this study have little to no spectral peaks above 300 Hz (Fig. 13), which explains why responses were obliterated when high-pass filtered at 300 Hz.

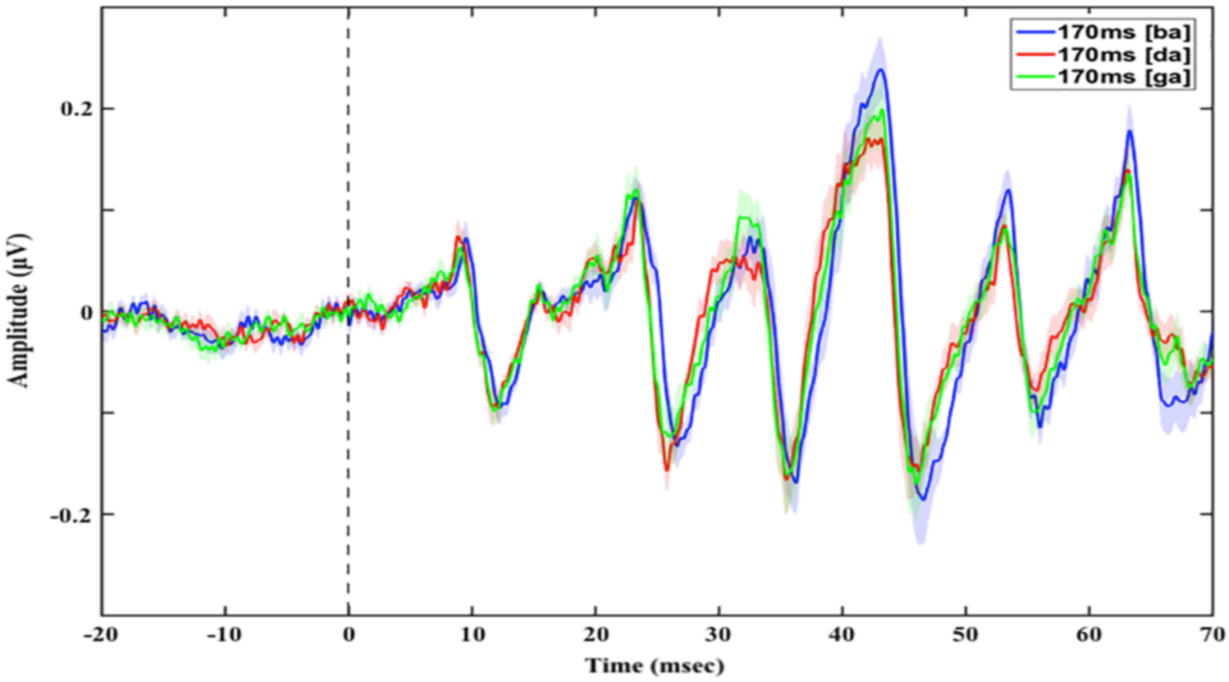

Fig. 9. Grand average speech-ABRs with pre-stimulus baseline (onset and transition period: 0 – 70ms) in quiet to the 170ms [ba] [da] [ga] overlaid, band-pass filtered 70 – 2000 Hz. Shade represents 1 SE.

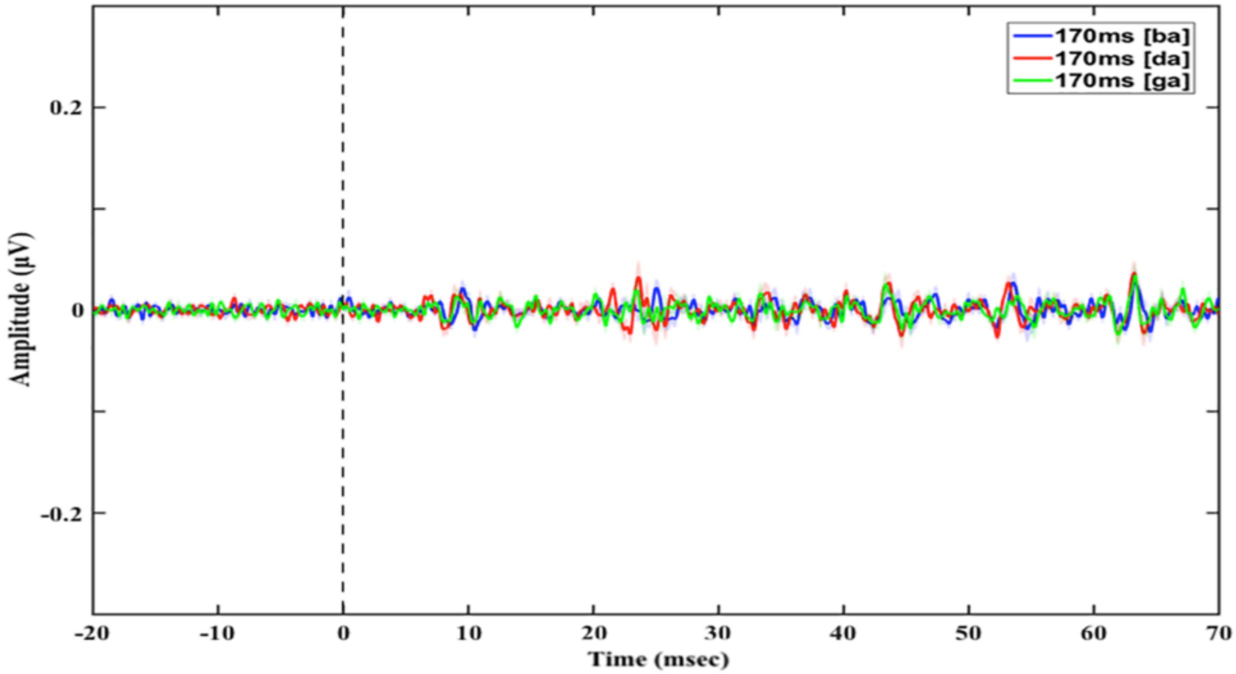

Fig. 10. Grand average speech-ABRs with pre-stimulus baseline (onset and transition period: 0 – 70ms) in quiet to the 170ms [ba] [da] [ga] overlaid, with additional high-pass filter (300 Hz) applied, showing the drastic decrease in amplitudes and overall absence of responses. Shade represents 1 SE

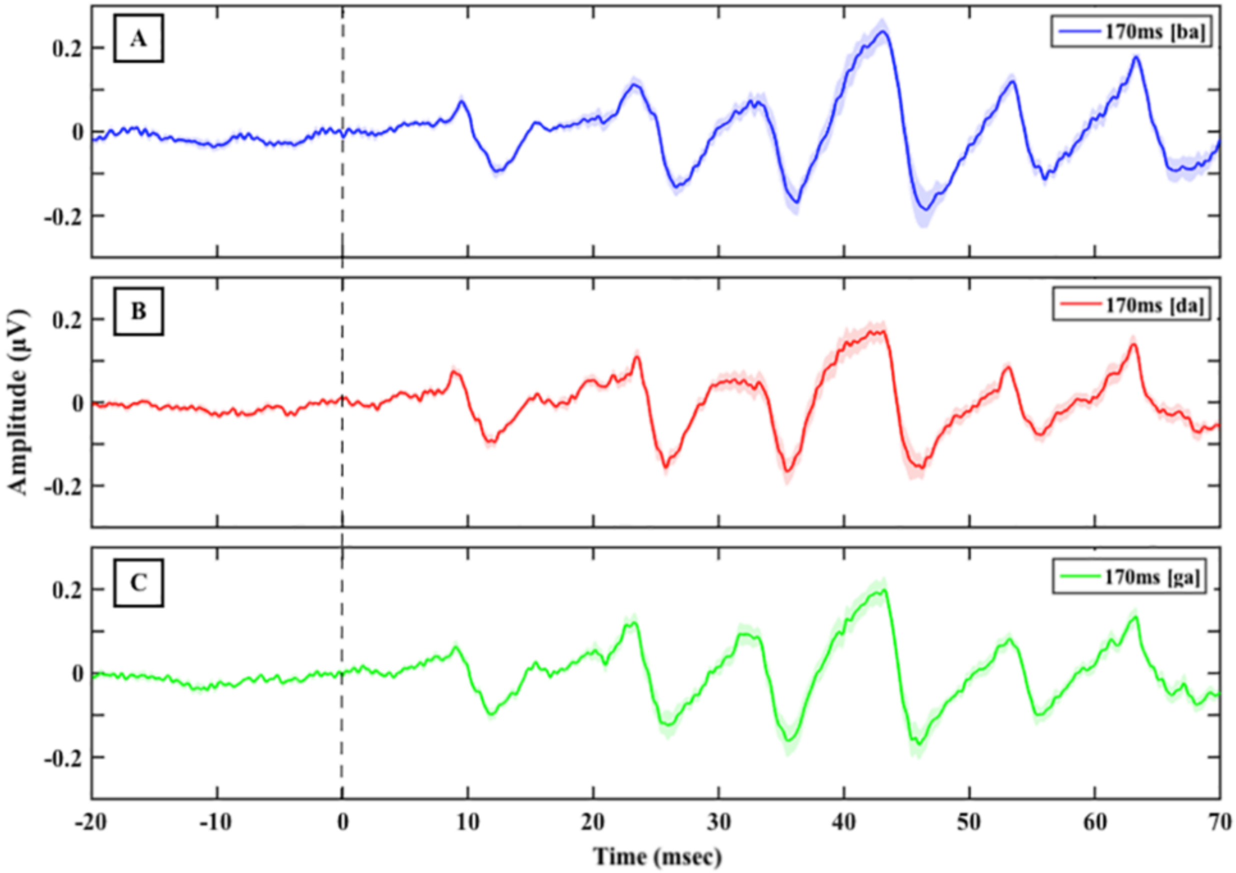

Fig. 11. Grand average speech-ABRs with pre-stimulus baseline (onset and transition period: 0 – 70ms) in quiet to the: (A) 170ms [ba], (B) 170ms [da], (C) 170ms [ga] plotted separately, band-pass filtered 70 – 2000 Hz. Shade in all panels represents 1 SE.

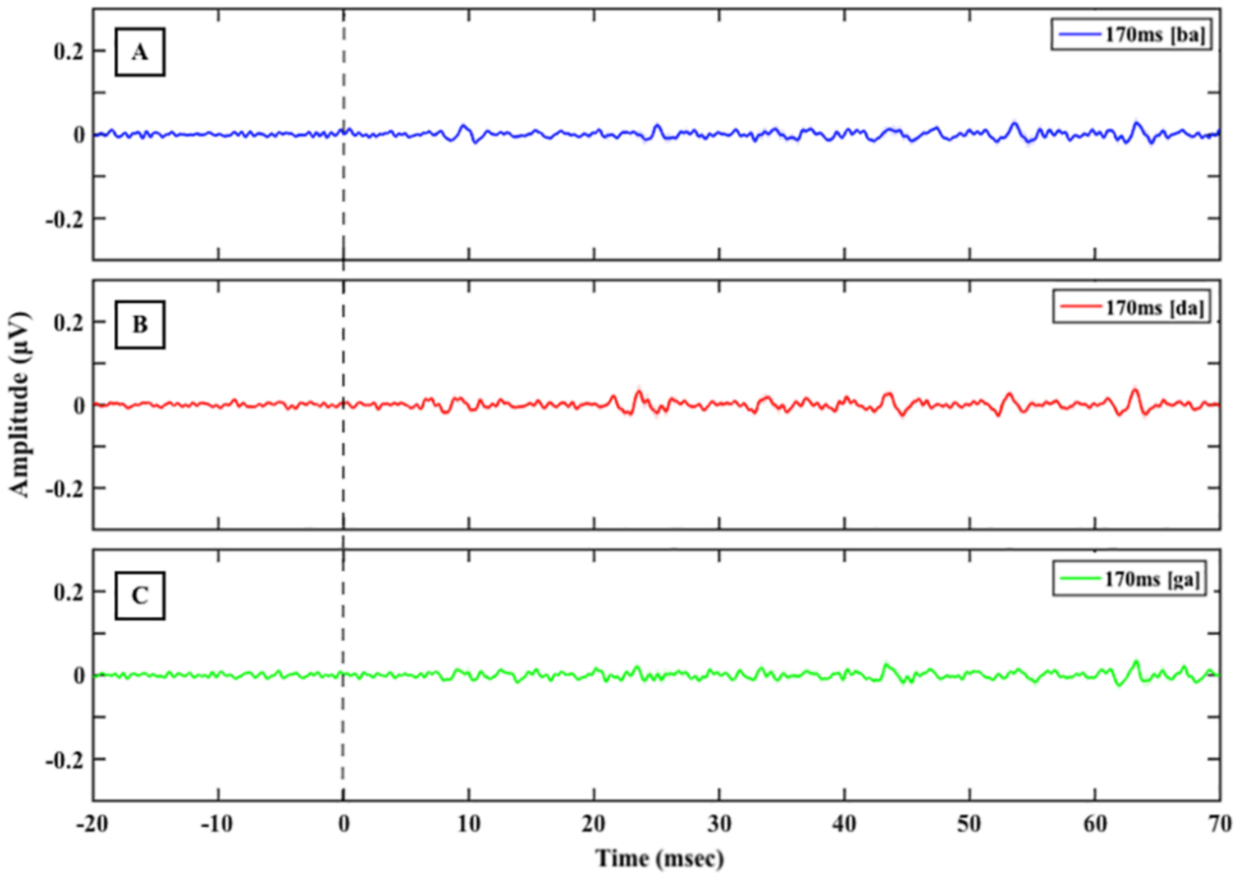

Fig. 12. Grand average speech-ABRs with pre-stimulus baseline (onset and transition period: 0 – 70ms) in quiet to the: (A) 170ms [ba], (B) 170ms [da], (C) 170ms [ga] plotted separately, with additional high-pass filter (300 Hz) applied. Shade in all panels represents 1 SE.

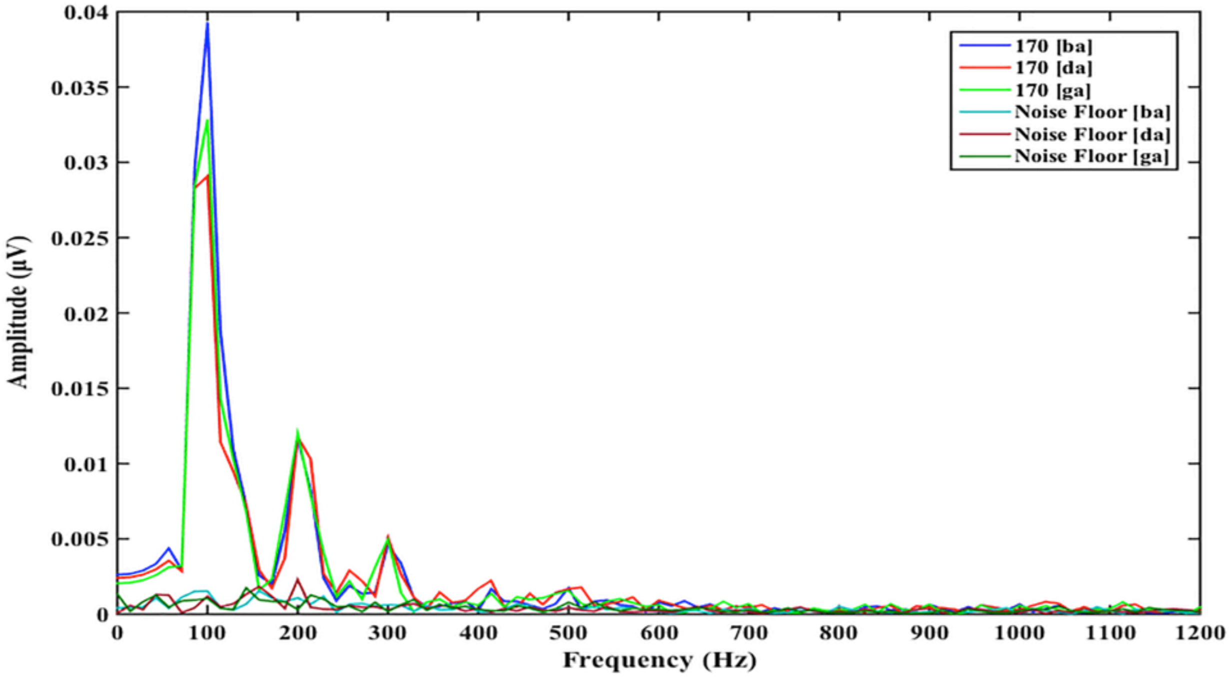

Fig. 13. Spectrum of grand average speech-ABRs band-pass filtered 70 – 2000 Hz (FFT of onset and transition period: 0 – 70ms) in quiet to the 170ms [ba], [da], and [ga] showing little to no spectral peaks above 300 Hz.

Section 4: Why Speech-ABRs Contained No Spectral Peaks Above 300 Hz

In order to best predict the expected spectra of the speech-ABRs, half-wave rectifying the acoustic signals of the 2 stimulus polarities then processing their waveforms through the same analyses as the speech-ABR raw data provides a prediction of the spectral characteristics of the speech-ABR in idealized circumstances (i.e. if the auditory system encodes the acoustic waveform with absolute accuracy). Therefore, for the acoustic stimulus spectra to be comparable to the speech-ABR spectra, the 170ms [ba] [da] [ga] acoustic stimuli were processed similarly to the speech-ABRs for comparison. The following steps were conducted:

1. For each stimulus (170ms [ba], 170ms [da], and 170ms [ga]), each stimulus polarity was half-wave rectified.
2. The half-wave rectified 2 polarities of each stimulus were added (as speech-ABRs to the 2 stimulus polarities were added).
3. FFTs were performed on the transition period (first 50ms) of the added half-wave rectified stimuli.

The resulting spectra of the half-wave rectified added stimuli (Fig. 14) are similar to the speech-ABR spectra, i.e. they contain 3 peaks at 100 Hz, 200 Hz, and 300 Hz and no clear spectral peaks above 300 Hz. It would therefore not be expected for the speech-ABRs to these stimuli to contain any spectral peaks above 300 Hz.

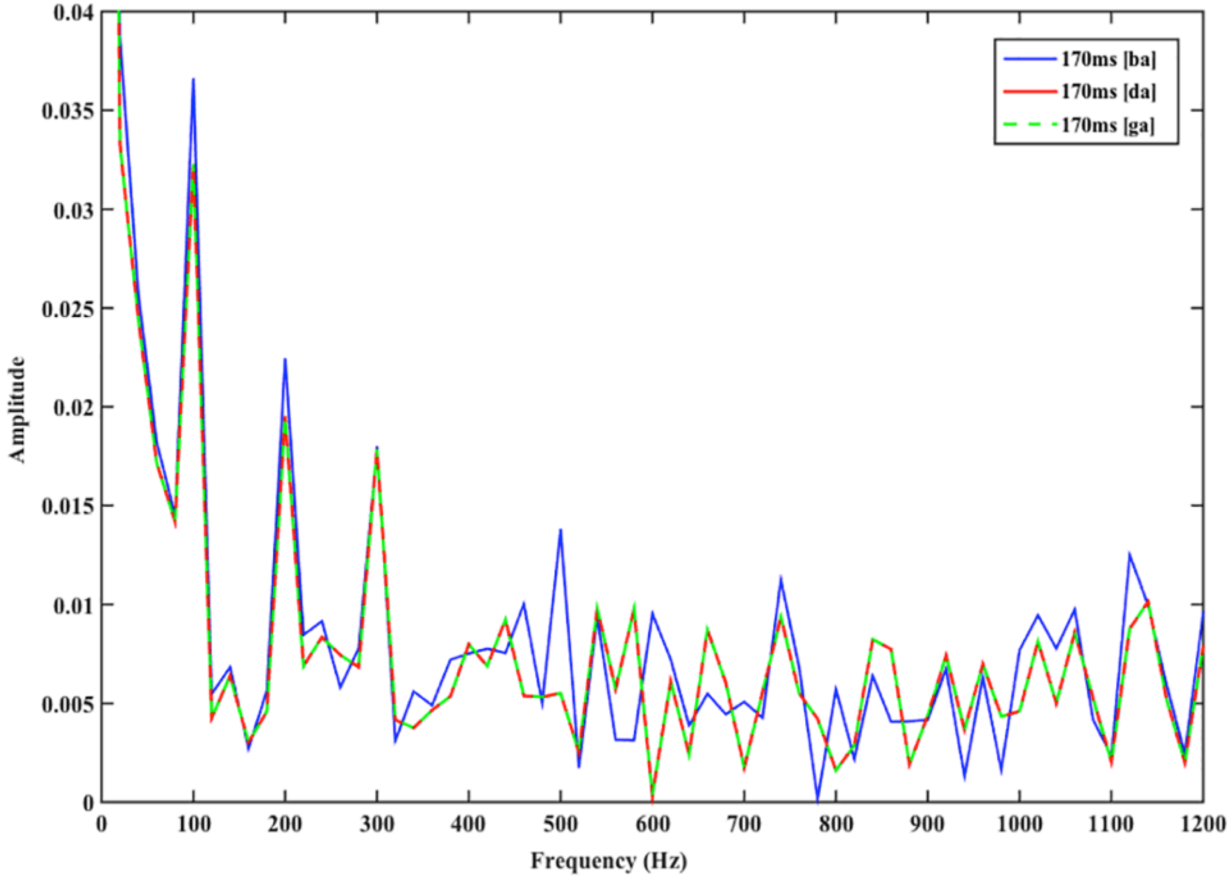

Fig. 14. Spectrum (FFT of the first 50ms) of the transition period of the half-wave rectified and added 170ms [ba] [da] and [ga] stimuli, showing 3 peaks at 100 Hz, 200 Hz, and 300 Hz with little to no spectral content above 300 Hz.

References:

Banai, K., Hornickel, J., Skoe, E., et al. (2009). Reading and subcortical auditory function. *Cerebral Cortex*, 19, 2699–2707.

Hornickel, J., Skoe, E., Nicol, T., et al. (2009). Subcortical differentiation of stop consonants relates to reading and speech-in-noise perception. *Proceedings of the National Academy of Sciences*, 106, 13022–13027.

Johnson, K., Nicol, T., Zecker, S., et al. (2008). Brainstem encoding of voiced consonant–vowel stop syllables. *Clinical Neurophysiology*, 119, 2623–2635.
