# Supplementary material for: Speech Auditory Brainstem Responses: Effects of Background, Stimulus Duration, Consonant–Vowel, and Number of Epochs
Source: Ear Hear. 2019 Apr 26;40(3):659–70. doi: 10.1097/AUD.0000000000000648 (PMC6493675; doi:10.1097/AUD.0000000000000648)
Supplement: Supplementary file 2 [file aud-40-659-s002.pdf]

Supplemental Digital Content 2

Speech Auditory Brainstem Responses: Effects of Background, Stimulus Duration, Consonant-Vowel, and Number of Epochs

Section 1: Detection of Speech-ABR Peaks

Table 1. Peaks that were missing in each participant's speech-ABRs in quiet (Q) and in noise (N) to all stimuli.

Shaded cells indicate no peaks were missing

	40ms [da]		50ms stimuli						170ms stimuli			
			[ba]		[da]		[ga]		[ba]	[da]		[ga]
**Participant**	**Q**	**N**	**Q**	**N**	**Q**	**N**	**Q**	**N**	**Q**	**Q**	**N**	**Q**
**1**		D, F										
**2**				O							V	
**3**		D		V, O			V	V, A			V	
**4**				V				V				
**5**				E				V			V, A	
**6**		F									V	
**7**			O	O		V		D				
**8**												
**9**	F	F										
**10**		F				V		V	V	V	V	V
**11**	F						D	D				
**12**			O	O								

Section 2: Speech-ABR Mean (SD) Peak Latencies and Amplitudes

Table 2. Mean and SD speech-ABR peak latency values (corrected for insert tube length) in quiet and in noise to the three [da] durations.

	40ms [da]				50ms [da]				170ms [da]			
	Quiet		Noise		Quiet		Noise		Quiet		Noise	
Peak	Mean	SD	Mean	SD	Mean	SD	Mean	SD	Mean	SD	Mean	SD
V	7.15	0.26	7.73	0.48	8.28	0.43	8.81	0.30	8.47	0.41	9.15	0.31
A	8.31	0.77	8.84	0.52	10.65	0.74	11.08	0.61	11.26	0.67	11.85	0.86
D	23.46	0.94	24.10	1.22	24.67	0.56	26.18	1.65	25.68	1.64	26.33	1.47
E	32.23	0.77	32.34	0.73	33.80	1.53	35.23	1.67	35.36	1.48	35.97	1.44
F	41.00	1.50	41.87	2.14	44.44	0.86	46.29	1.76	45.65	1.75	46.53	1.67
O	48.68	0.38	48.96	0.45	55.44	1.79	56.45	1.37	55.78	1.68	57.39	2.15

Table 3. Mean and SD speech-ABRs peak latency values (corrected for insert tube length) to the 50ms [ba] and [ga] in quiet and in noise, and to the 170ms [ba] and [ga] in quiet.

	50ms [ba]				170ms [ba]		50ms [ga]				170ms [ga]	
	Quiet		Noise		Quiet		Quiet		Noise		Quiet	
Peak	Mean	SD	Mean	SD	Mean	SD	Mean	SD	Mean	SD	Mean	SD
V	8.20	0.46	8.91	0.46	8.80	0.42	8.39	0.49	8.94	0.70	8.68	0.44
A	10.42	0.75	11.16	0.59	11.55	0.81	10.96	0.65	11.39	0.74	11.00	0.81
D	25.41	1.91	26.96	1.91	25.91	0.73	24.26	1.59	26.03	1.43	25.15	1.02
E	34.77	3.25	36.50	4.82	35.32	1.24	33.96	0.90	34.45	1.14	35.17	1.70
F	44.84	3.33	46.47	3.63	46.10	0.95	44.54	0.76	45.19	0.92	45.22	0.75
O	55.18	1.40	57.17	1.78	55.43	1.17	55.40	1.73	55.84	1.62	55.45	1.52

Table 4. Mean and SD speech-ABR peak amplitude values in quiet and in noise to the three [da] durations.

	**40ms [da]**				**50ms [da]**				**170ms [da]**			
	**Quiet**		**Noise**		**Quiet**		**Noise**		**Quiet**		**Noise**	
**Peak**	**Mean**	**SD**	**Mean**	**SD**	**Mean**	**SD**	**Mean**	**SD**	**Mean**	**SD**	**Mean**	**SD**
**VA**	0.32	0.11	0.18	0.06	0.25	0.09	0.14	0.05	0.23	0.09	0.11	0.05
**D**	0.31	0.15	0.20	0.18	0.28	0.10	0.22	0.09	0.34	0.12	0.23	0.07
**E**	0.34	0.11	0.21	0.06	0.28	0.07	0.19	0.07	0.36	0.10	0.21	0.07
**F**	0.23	0.10	0.16	0.13	0.42	0.10	0.34	0.09	0.47	0.16	0.30	0.06
**O**	0.32	0.09	0.27	0.09	0.24	0.10	0.21	0.08	0.26	0.07	0.23	0.09

Table 5. Mean and SD speech-ABR peak amplitude values to the 50ms [ba] and [ga] in quiet and in noise, and to the 170ms [ba] and [ga] in quiet.

	**50ms [ba]**				**170ms [ba]**		**50ms [ga]**				**170ms [ga]**	
	**Quiet**		**Noise**		**Quiet**		**Quiet**		**Noise**		**Quiet**	
**Peak**	**Mean**	**SD**	**Mean**	**SD**	**Mean**	**SD**	**Mean**	**SD**	**Mean**	**SD**	**Mean**	**SD**
**VA**	0.24	0.07	0.14	0.04	0.23	0.08	0.24	0.07	0.14	0.06	0.22	0.07
**D**	0.25	0.09	0.18	0.06	0.33	0.10	0.27	0.13	0.16	0.09	0.36	0.13
**E**	0.36	0.25	0.17	0.10	0.38	0.09	0.34	0.11	0.20	0.08	0.38	0.10
**F**	0.44	0.14	0.32	0.11	0.55	0.22	0.41	0.10	0.31	0.09	0.49	0.17
**O**	0.24	0.10	0.17	0.14	0.30	0.10	0.27	0.07	0.31	0.16	0.27	0.09

Section 3: Effects of Background on Speech-ABRs – Post Hoc Pairwise Comparison Results

Table 6. Post hoc pairwise comparisons of speech-ABR peak latencies comparing the two backgrounds: quiet (Q) versus noise (N) per stimulus duration, showing differences in peak latencies in quiet versus in noise (Q minus N), standard error (SE), degrees of freedom (df), *t* ratio, and bonferroni corrected *p* values.

Significant *p* values are shown in **blue**

	**Stimulus duration: 40ms**					**Stimulus duration: 50ms**					**Stimulus duration: 170ms**				
**Peak**	**Q – N (ms)**	**SE**	**df**	***t* ratio**	***p***	**Q – N (ms)**	**SE**	**df**	***t* ratio**	***p***	**Q – N (ms)**	**SE**	**df**	***t* ratio**	***p***
**V**	-0.91	0.10	796.07	-9.42	**0.0054**	-0.91	0.10	796.07	-9.42	**0.0054**	-0.91	0.10	796.07	-9.42	**0.0054**
**A**	-0.91	0.10	796.07	-9.42	**0.0054**	-0.91	0.10	796.07	-9.42	**0.0054**	-0.91	0.10	796.07	-9.42	**0.0054**
**D**	-0.91	0.10	796.07	-9.42	**0.0054**	-0.91	0.10	796.07	-9.42	**0.0054**	-0.91	0.10	796.07	-9.42	**0.0054**
**E**	-0.91	0.10	796.07	-9.42	**0.0054**	-0.91	0.10	796.07	-9.42	**0.0054**	-0.91	0.10	796.07	-9.42	**0.0054**
**F**	-0.91	0.10	796.07	-9.42	**0.0054**	-0.91	0.10	796.07	-9.42	**0.0054**	-0.91	0.10	796.07	-9.42	**0.0054**
**O**	-0.91	0.10	796.07	-9.42	**0.0054**	-0.91	0.10	796.07	-9.42	**0.0054**	-0.91	0.10	796.07	-9.42	**0.0054**

Table 7. Post hoc pairwise comparisons of Speech-ABR peak amplitudes comparing the two backgrounds: quiet (Q) versus noise (N) per stimulus duration, showing differences in peak amplitudes in quiet versus in noise (Q minus N), standard error (SE), degrees of freedom (df), *t* ratio, and bonferroni corrected *p* values.

Significant *p* values are shown in **blue**

	**Stimulus duration: 40ms**					**Stimulus duration: 50ms**					**Stimulus duration: 170ms**				
**Peak**	**Q – N (µV)**	**SE**	**df**	***t* ratio**	***p***	**Q – N (µV)**	**SE**	**df**	***t* ratio**	***p***	**Q – N (µV)**	**SE**	**df**	***t* ratio**	***p***
**VA**	0.11	0.02	687	5.06	**0.0045**	0.10	0.02	687	5.73	**0.0045**	0.14	0.02	687	7.21	**0.0045**
**D**	0.09	0.02	687	4.19	**0.0045**	0.08	0.02	687	4.62	**0.0045**	0.12	0.02	687	6.24	**0.0045**
**E**	0.14	0.02	687	6.47	**0.0045**	0.13	0.02	687	7.54	**0.0045**	0.17	0.02	687	8.78	**0.0045**
**F**	0.12	0.02	687	5.68	**0.0045**	0.11	0.02	687	6.53	**0.0045**	0.15	0.02	687	7.91	**0.0045**
**O**	0.03	0.02	687	1.34	0.1823	0.02	0.02	687	0.98	0.3263	0.06	0.02	687	3.07	0.1035

Section 4: Bootstrap Results and Examples

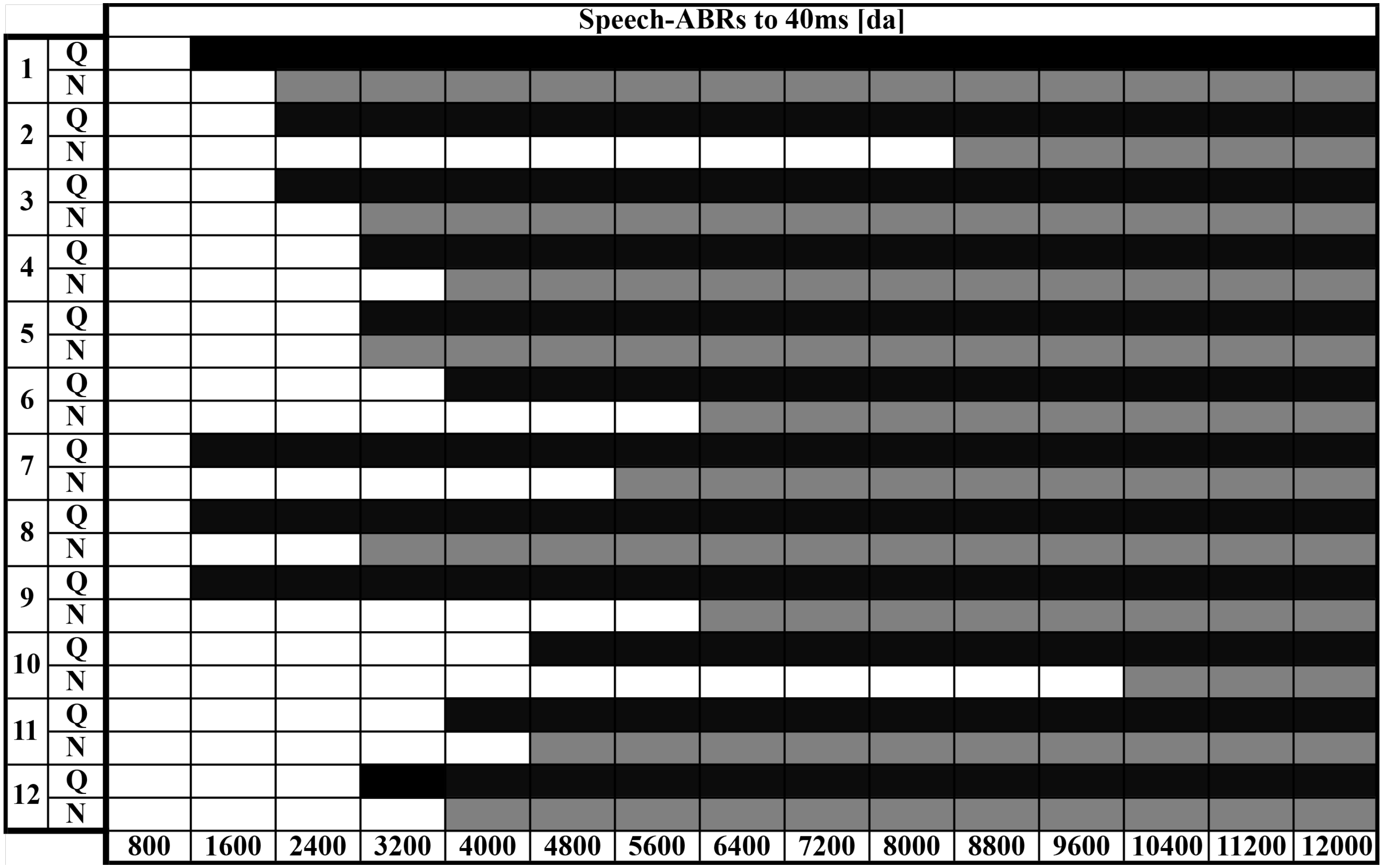

Fig. 1. Speech-ABRs to the 40ms [da] in quiet (black) and noise (grey) at 15 iterations per participant: shaded cells indicate that $F_{SP} \geq 3.1$ and all peaks that were detected at 12000 epochs were detected with 95% confidence via bootstrap. White cells indicate that not all peaks were detected with 95% confidence via bootstrap.

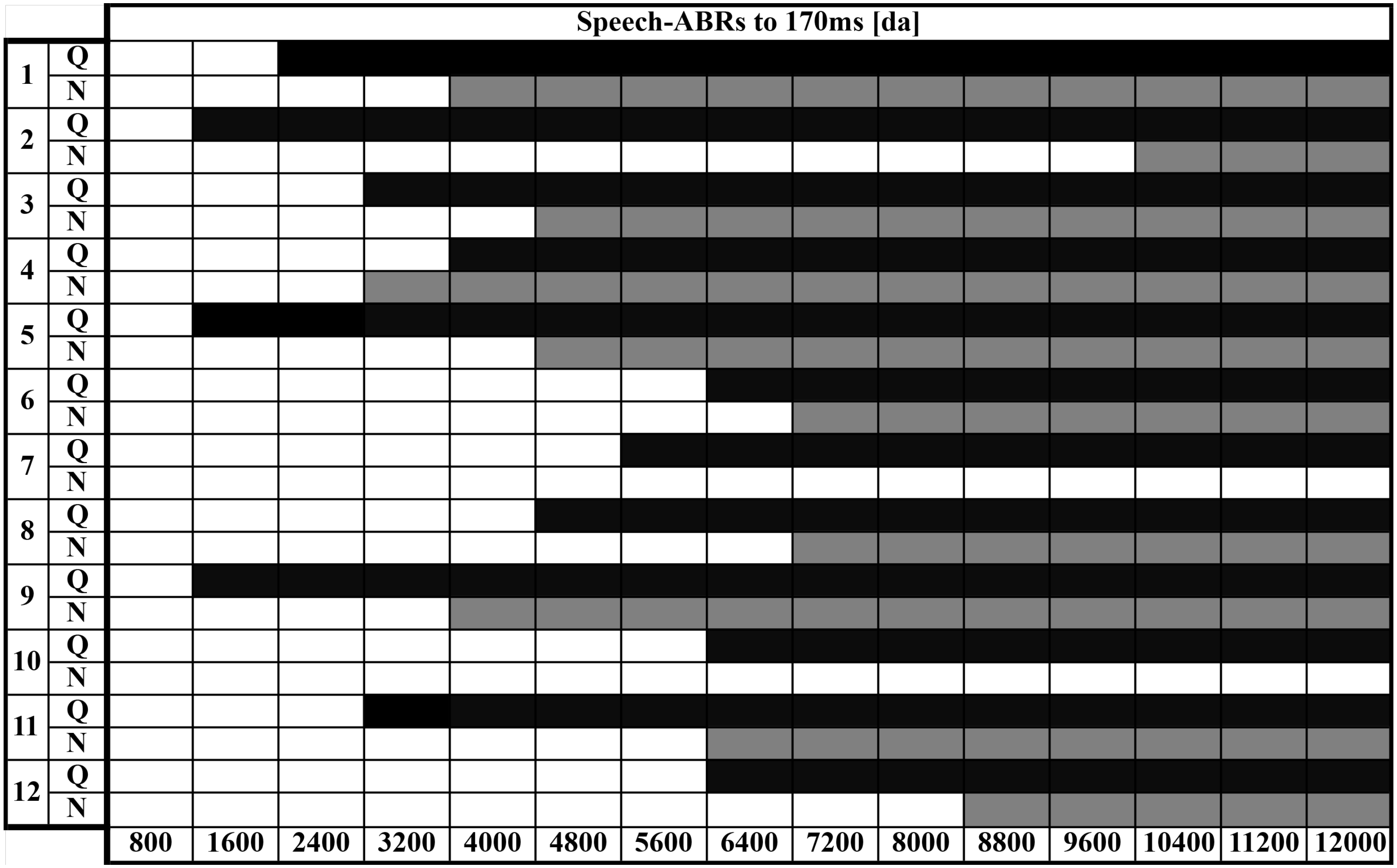

Fig. 2. Speech-ABRs to the 170ms [da] in quiet (black) and noise (grey) at 15 iterations per participant: shaded cells indicate that $F_{SP} \geq 3.1$ and all peaks that were detected at 12000 epochs were be detected with 95% confidence via bootstrap. White cells indicate that not all peaks were detected with 95% confidence via bootstrap.

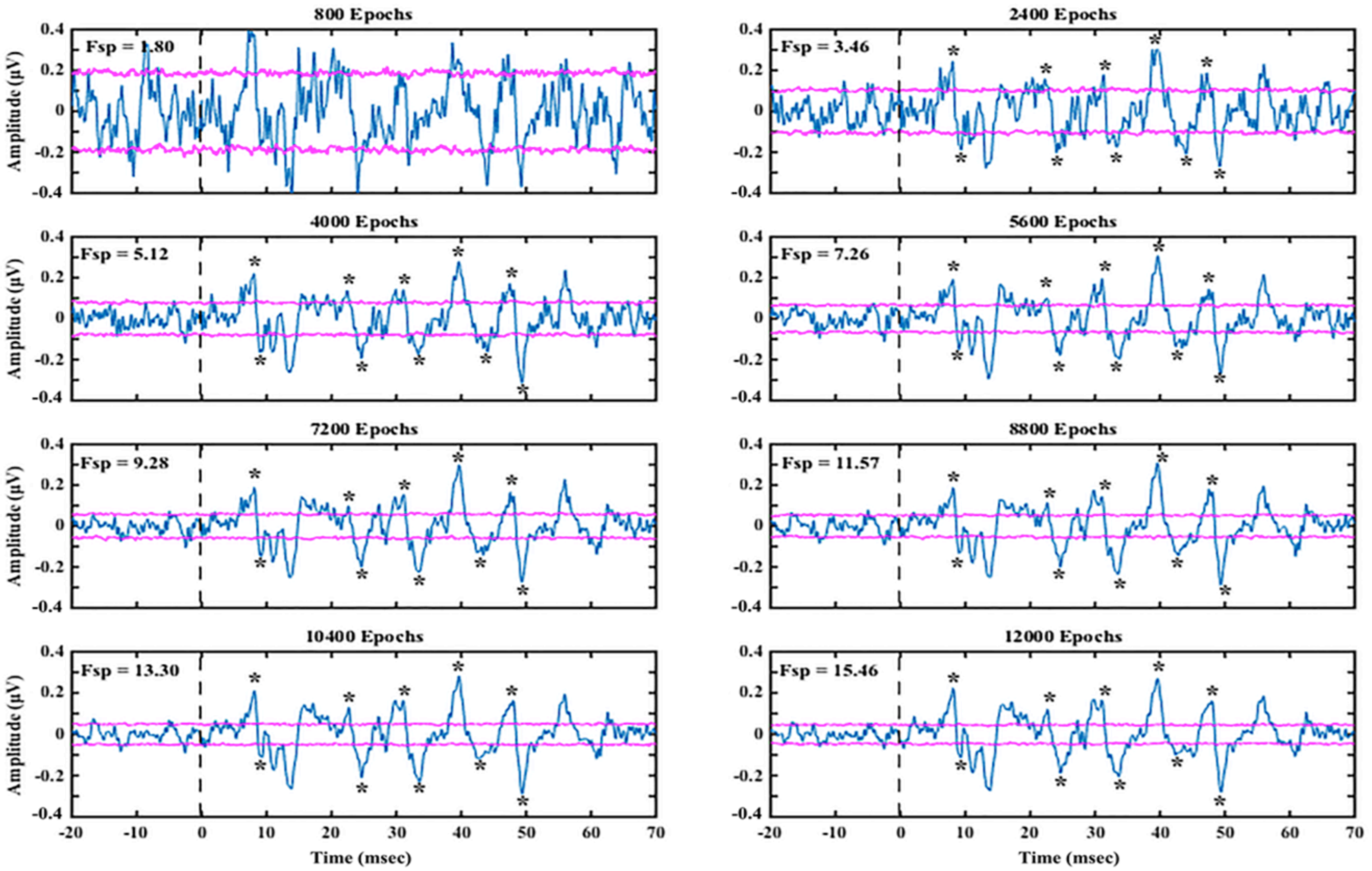

Fig. 3. Speech-ABRs with pre-stimulus baseline to the 40ms [da] in quiet at 8 iterations from a participant (5) with better responses. Peaks that were detected with 95% confidence once F_{SP} reached $\geq$ 3.1 are marked with a '*'.

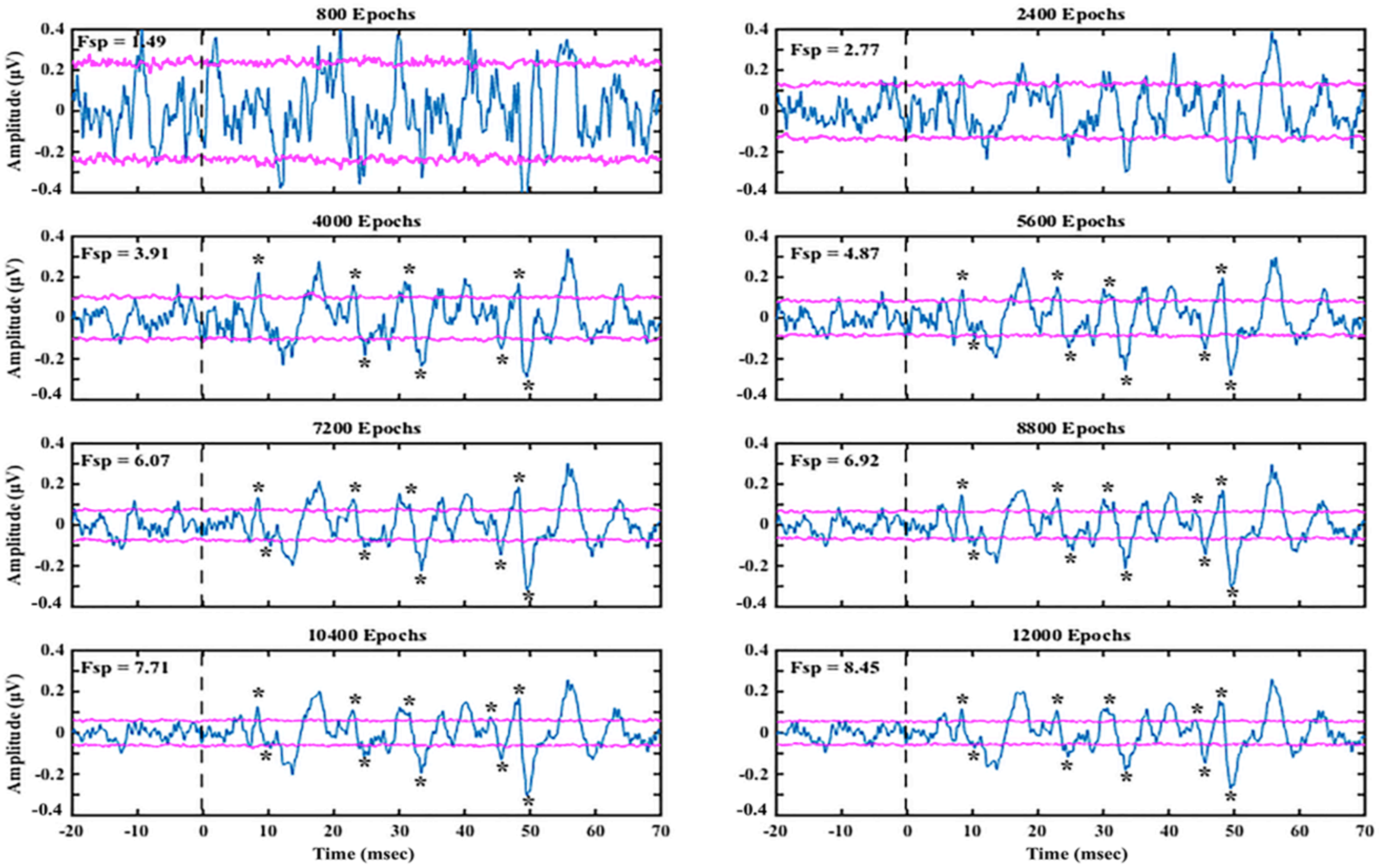

Fig. 4. Speech-ABRs with pre-stimulus baseline to the 40ms [da] in noise at 8 iterations from a participant (5) with better responses. Peaks that were detected with 95% confidence once F_{SP} reached ≥ 3.1 are marked with a '*'.

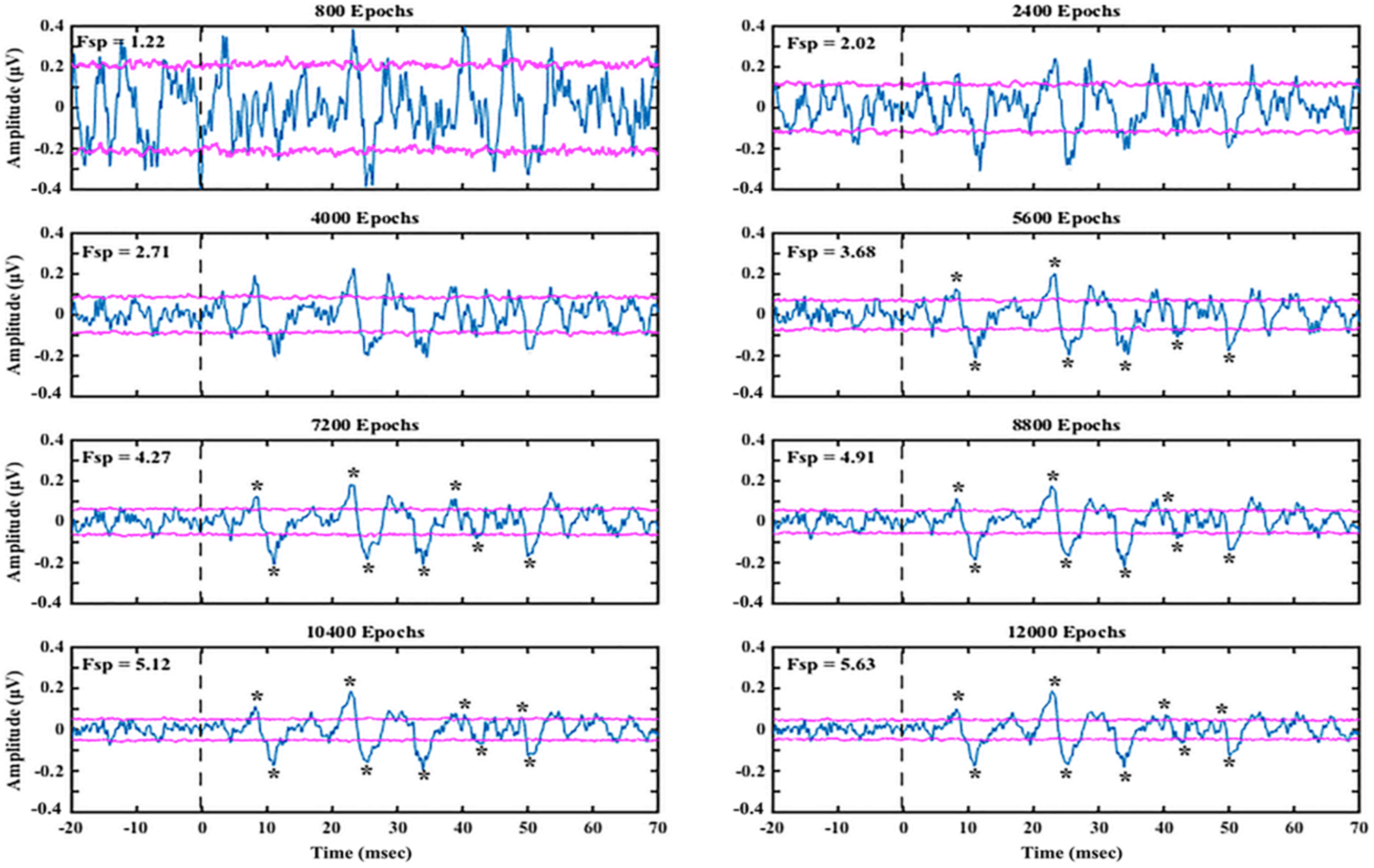

Fig. 5. Speech-ABRs with pre-stimulus baseline to the 40ms [da] in quiet at 8 iterations from a participant (10) with poorer responses. Peaks that were detected with 95% confidence once F_{SP} reached $\geq$ 3.1 are marked with a '*'.

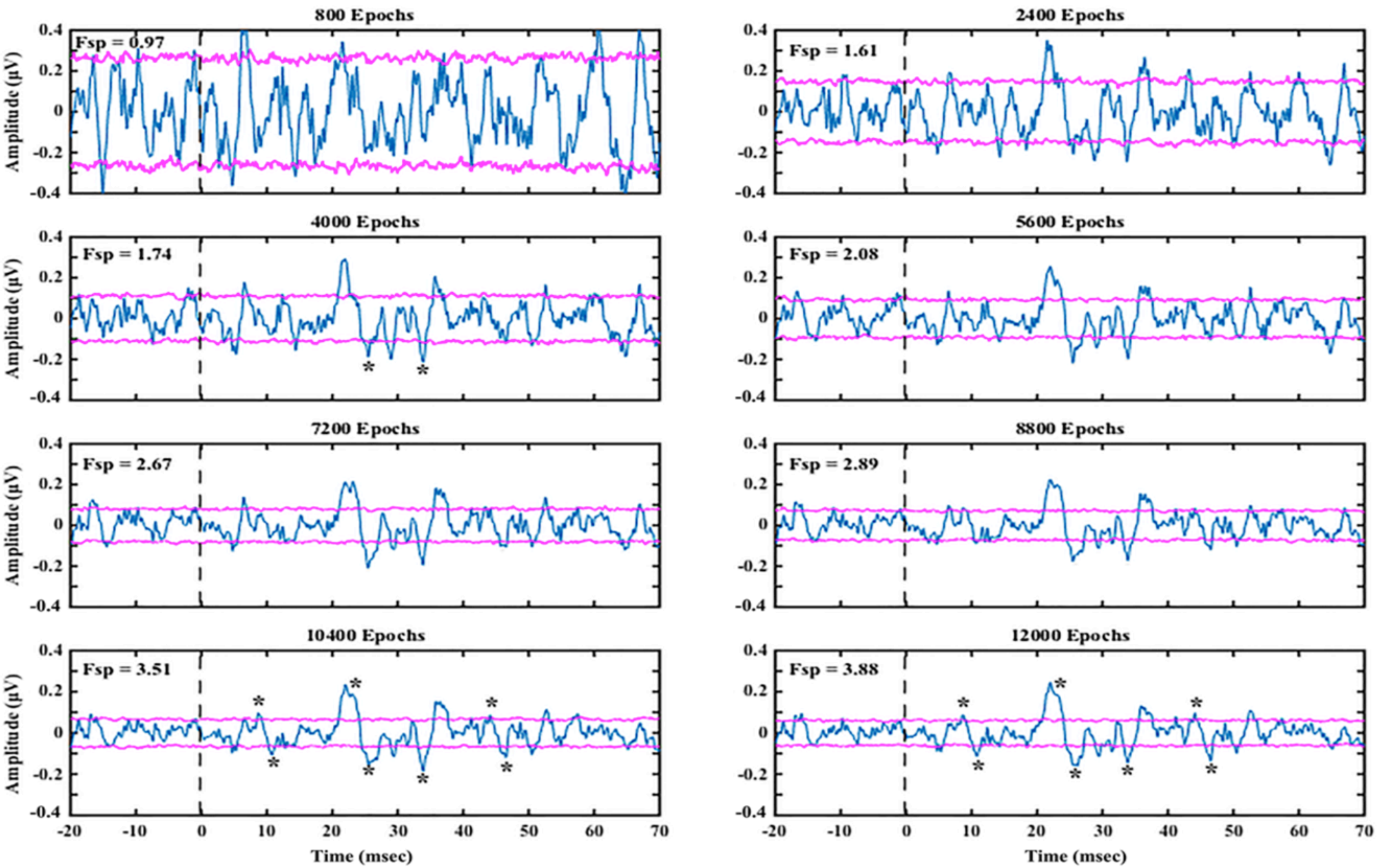

Fig. 6. Speech-ABRs with pre-stimulus baseline to the 40ms [da] in noise at 8 iterations from a participant (10) with poorer responses. Peaks that were detected with 95% confidence once F_{SP} reached $\geq$ 3.1 are marked with a '*'.

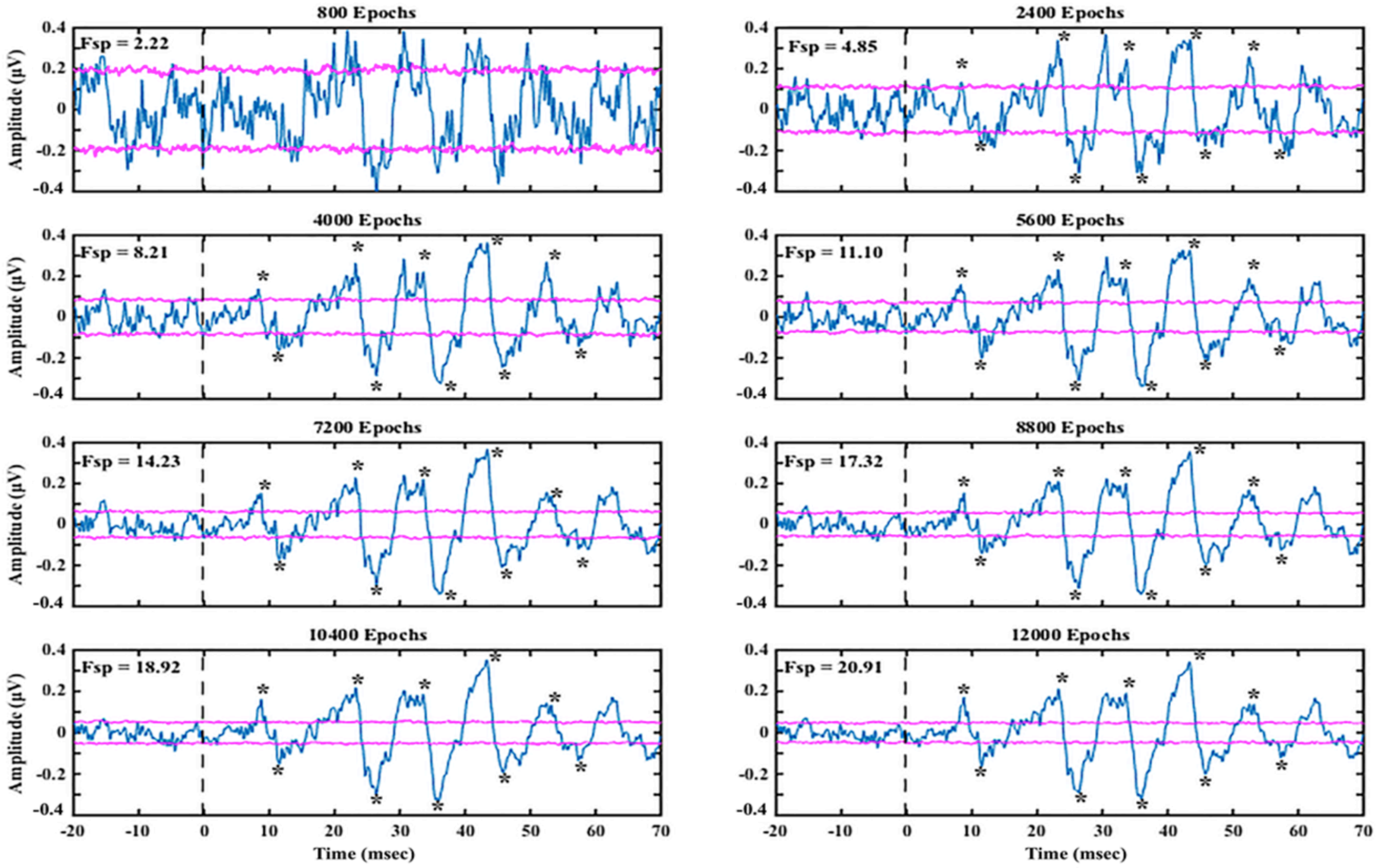

Fig. 7. Speech-ABRs with pre-stimulus baseline to the 170ms [da] in quiet at 8 iterations from a participant (5) with better responses. Peaks that were detected with 95% confidence once F_{SP} reached ≥ 3.1 are marked with a '*'.

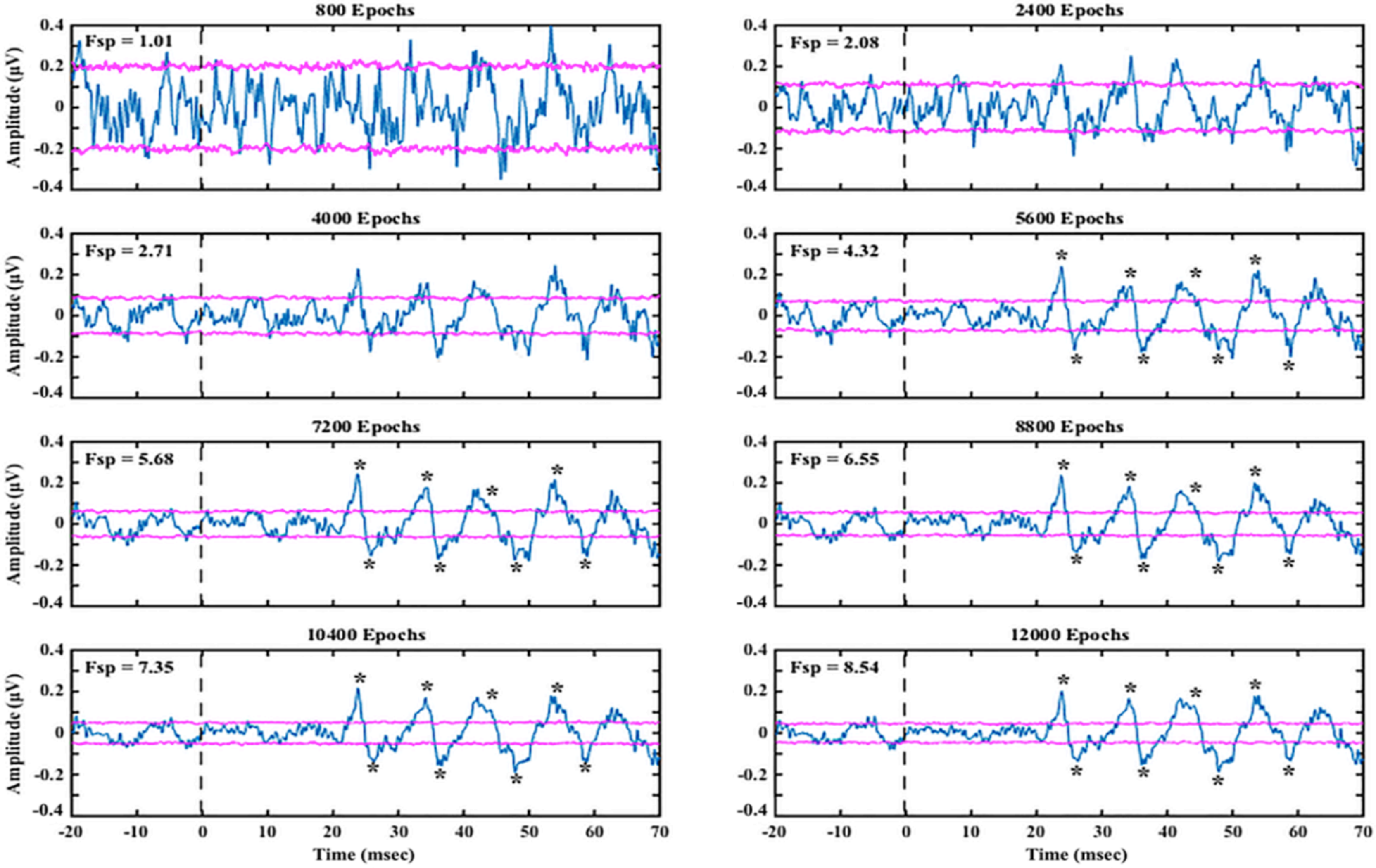

Fig. 8. Speech-ABRs with pre-stimulus baseline to the 170ms [da] in noise at 8 iterations from a participant (5) with better responses. Peaks that were detected with 95% confidence once F_{SP} reached $\geq$ 3.1 are marked with a '*'.

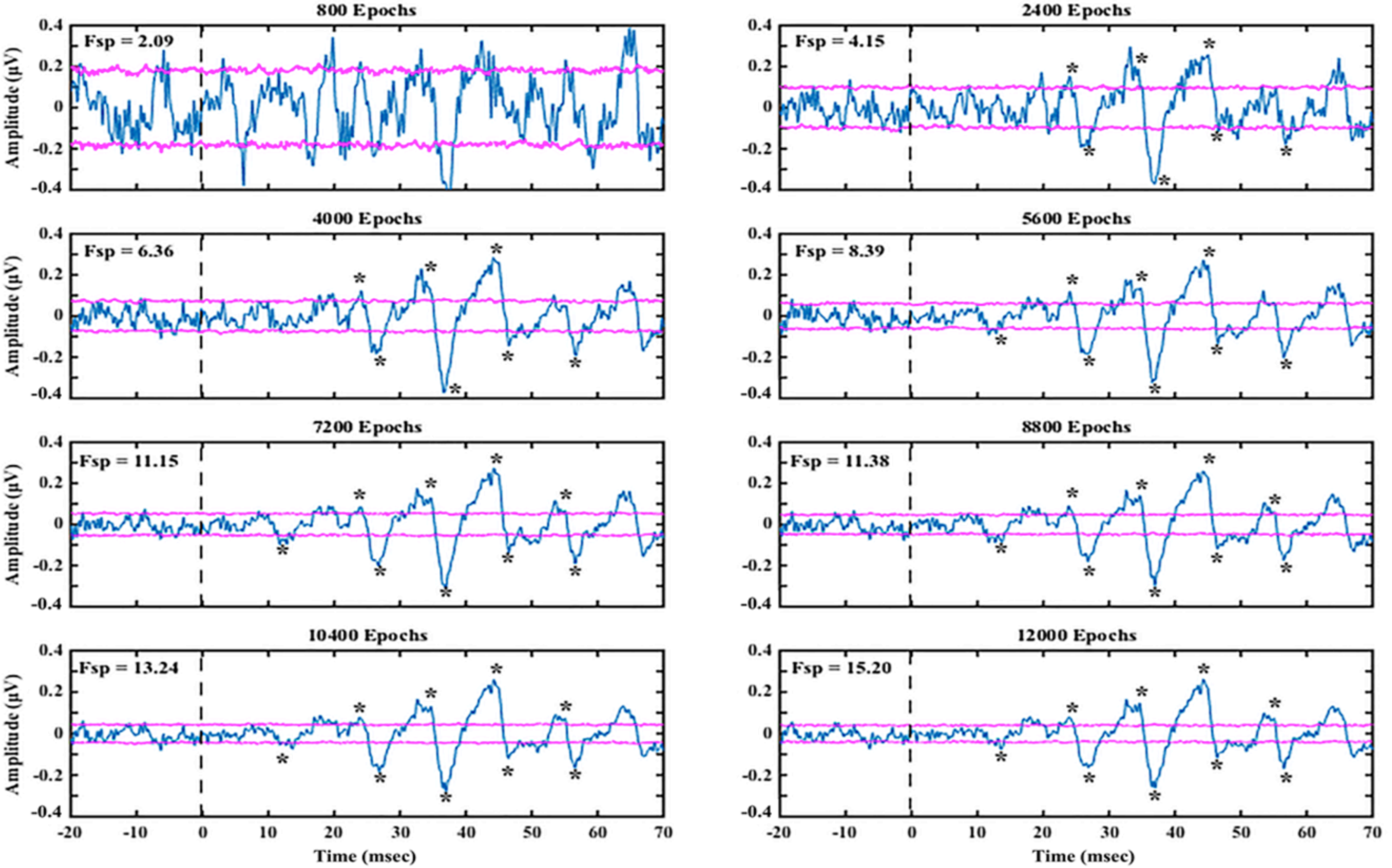

Fig. 9. Speech-ABRs with pre-stimulus baseline to the 170ms [da] in quiet at 8 iterations from a participant (10) with poorer responses. Peaks that were detected with 95% confidence once F_{SP} reached ≥ 3.1 are marked with a '*'.

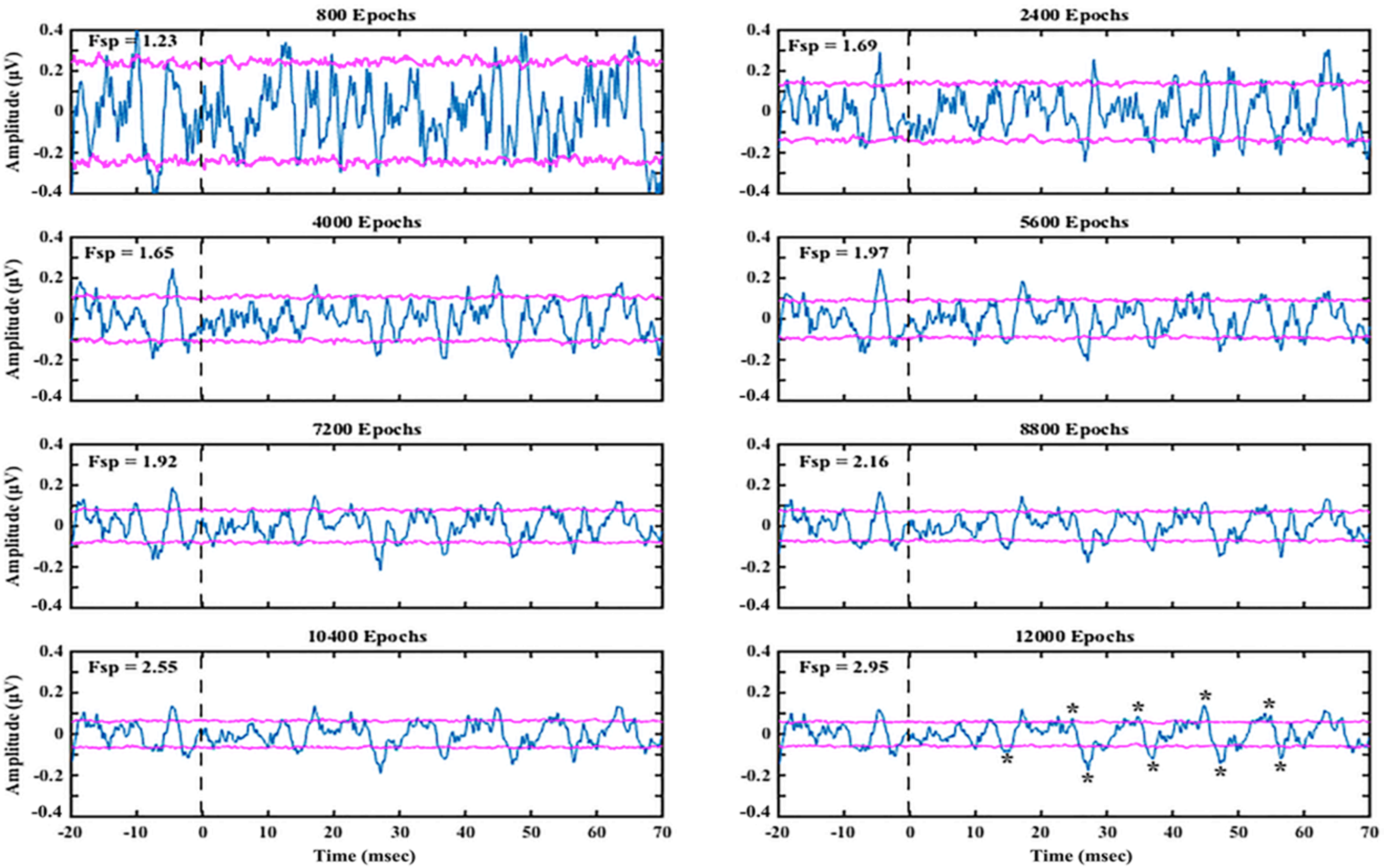

Fig. 10. Speech-ABRs with pre-stimulus baseline to the 170ms [da] in noise at 8 iterations from a participant (10) with poorer responses. F_{SP} did not reach 3.1 in this participant to the 170ms [da] in noise, therefore peaks that were detected with 95% confidence are only marked with a '*' at 12000 epochs.

Section 5: F_{SP} Values And Number of Epochs

Table 8. Number of epochs where $F_{SP} \geq 3.1$ for speech-ABRs to 40ms and 170ms [da] in quiet and in noise (per participant), F_{SP} value, difference in number of epochs between quiet and noise (diff).

Blank cells shaded in red indicate that participant speech-ABRs did not reach $F_{SP} \geq 3.1$.

	40ms [da]					170ms [da]				
	Quiet		Noise		Diff	Quiet		Noise		Diff
	Epochs	F_{SP}	Epochs	F_{SP}		Epochs	F_{SP}	Epochs	F_{SP}	
**1**	1600	3.37	2400	4.46	800	800	4.88	1600	3.28	800
**2**	2400	4.72	4800	3.83	2400	1600	3.40	5600	3.22	4000
**3**	2400	4.23	3200	3.34	800	3200	3.63	4000	3.23	800
**4**	2400	4.35	2400	3.79	0	1600	4.65	2400	3.88	800
**5**	2400	3.46	3200	3.58	800	1600	4.15	4800	3.50	3200
**6**	3200	3.50	6400	3.35	3200	1600	3.41	7200	3.44	5600
**7**	1600	3.50	5600	3.14	4000	2400	3.67			
**8**	1600	6.17	1600	3.90	0	1600	3.19	2400	3.99	800
**9**	1600	3.47	2400	3.13	800	1600	3.25	4000	3.21	2400
**10**	4800	3.16	9600	3.25	4800	2400	4.15			
**11**	3200	3.26	4800	3.42	1600	3200	3.32	6400	3.27	3200
**12**	3200	3.54	4000	3.25	800	4000	3.47	8800	3.26	4800
**Mean**	**2533.33**	**3.89**	**4200**	**3.54**	**1666.67**	**2133.33**	**3.76**	**4720**	**3.43**	**2181.82**
**SD**	**954.73**	**0.86**	**2240.13**	**0.39**	**1580.18**	**923.76**	**0.56**	**2307.86**	**0.28**	**2293.39**

Table 9. Number of epochs (at or above epochs required for $F_{SP} \geq 3.1$) where peaks were detected with 95% confidence via bootstrap for speech-ABRs to 40ms and 170ms [da] in quiet and in noise (per participant), F_{SP} values, difference in number of epochs between quiet and noise (diff).
* A larger number of epochs than required to reach $F_{sp} > 3.1$ was required to detect all peaks.
Blank cells shaded in red indicate that participant speech-ABRs did not reach $F_{SP} > 3.1$.

	40ms [da]					170ms [da]				
	Quiet		Noise		Diff	Quiet		Noise		Diff
	Epochs	F_{SP}	Epochs	F_{SP}		Epochs	F_{SP}	Epochs	F_{SP}	
**1**	1600	3.37	2400	4.46	800	2400*	12.42	4000*	6.82	1600
**2**	2400	4.72	8800*	6.16	6400	1600	3.40	10400*	4.75	8800
**3**	2400	4.23	3200	3.34	800	3200	3.63	4800*	3.71	1600
**4**	3200*	5.46	4000*	5.07	800	4000*	10.34	3200*	4.84	-800
**5**	2400	4.28	3200	3.58	800	1600	4.15	4800	3.5	3200
**6**	4000*	4.12	6400	3.35	2400	6400*	11.5	7200	3.44	800
**7**	1600	3.50	5600	3.14	4000	5600*	6.36			
**8**	1600	6.17	3200*	5.56	1600	4800*	10.51	7200*	8.86	2400
**9**	1600	3.47	6400*	5.98	4800	1600	3.25	4000	3.21	2400
**10**	4800	3.16	10400*	3.51	5600	6400*	9.73			
**11**	4000*	3.99	4800	3.42	800	3200	3.32	6400	3.27	3200
**12**	3200	3.54	4000	3.25	800	6400*	4.66	8800	3.26	2400
**Mean**	**2733.33**	**4.17**	**5200.00**	**4.24**	**2466.67**	**3933.33**	**6.94**	**7066.67**	**4.30**	**3133.33**
**SD**	**1103.16**	**0.91**	**2448.00**	**1.15**	**2142.78**	**1943.44**	**3.65**	**3123.32**	**1.82**	**2630.36**

Table 10. F_{sp} values for speech-ABRs to the three [da] durations in quiet and in noise (per participant) at 12000 epochs and 'no sound' F_{sp} values (per participant).

F_{sp} values in red are those below 3.1

	**40ms [da]**		**50ms [da]**		**170ms [da]**		**No Sound**
	**Quiet**	**Noise**	**Quiet**	**Noise**	**Quiet**	**Noise**	
**1**	23.69	19.00	23.06	18.63	22.32	17.83	1.05
**2**	15.84	9.15	22.41	4.47	10.74	5.09	0.66
**3**	16.00	9.30	8.34	13.41	8.22	11.42	0.82
**4**	20.87	11.96	17.88	13.70	5.51	13.83	1.27
**5**	15.46	8.45	7.86	5.58	10.94	8.54	0.70
**6**	10.99	5.01	13.93	5.22	7.29	5.15	1.02
**7**	20.04	6.95	9.82	3.46	4.64	**2.96**	0.76
**8**	36.69	19.99	21.96	11.38	11.64	14.45	1.05
**9**	25.71	10.04	16.67	9.62	8.48	8.81	1.46
**10**	5.63	3.88	6.76	4.29	6.37	**2.95**	0.72
**11**	11.12	5.86	3.68	**2.76**	5.45	5.34	0.87
**12**	12.84	8.14	9.36	4.24	3.60	4.28	1.09
**Mean**	**17.91**	**9.81**	**13.48**	**8.06**	**8.77**	**8.39**	**0.95**
**SD**	**8.23**	**5.05**	**6.76**	**5.14**	**4.99**	**4.97**	**0.25**

Table 11. F_{sp} values for speech-ABRs (per participant) to the 50ms [ba] and [ga] in quiet and in noise and to the 170ms [ba] and [ga] in quiet at 12000 epochs.

F_{sp} values in red are those below 3.1

	50ms [ba]		170ms [ba]	50ms [ga]		170ms [ga]
	**Quiet**	**Noise**	**Quiet**	**Quiet**	**Noise**	**Quiet**
**1**	50.91	24.51	48.21	18.15	8.85	29.36
**2**	8.11	**3.06**	29.92	5.92	**1.79**	31.24
**3**	11.05	3.27	16.12	4.17	**2.83**	10.82
**4**	8.81	7.55	28.48	11.68	10.98	25.38
**5**	11.55	4.61	21.60	8.88	4.95	25.37
**6**	13.47	4.31	17.36	11.90	5.09	13.90
**7**	9.89	7.03	12.22	10.60	3.98	15.74
**8**	29.63	13.60	24.38	20.99	11.21	19.36
**9**	17.48	12.44	37.67	19.73	8.78	41.69
**10**	7.19	**2.37**	8.52	6.77	3.56	12.66
**11**	6.64	4.12	6.92	5.38	3.44	7.38
**12**	6.42	4.36	15.32	11.25	2.86	12.76
**Mean**	**15.10**	**7.60**	**22.23**	**11.29**	**5.69**	**20.47**
**SD**	**12.99**	**6.43**	**12.24**	**5.67**	**3.34**	**10.20**
